# Linking movement-related beta oscillations to cortical excitability, structural damage, and fatigue in multiple sclerosis

**DOI:** 10.1093/braincomms/fcag043

**Published:** 2026-03-12

**Authors:** Elisa Tatti, Alberto Benelli, Alessandra Cinti, Rosa Cortese, Anna Serbina, Anna J Kulapurathazhe, Sophia Saed, Ludovico Luchetti, Javier Cudeiro, Jian Zhang, Anna de Mauro, Francesco Neri, Maria Laura Stromillo, Tommaso Lisini Baldi, Nicole d'Aurizio, Marco Battaglini, Domenico Plantone, Delia Righi, Elisa Massucco, Alessandro Giannotta, Francesco Lomi, Adriano Scoccia, Giuseppe Lai, Monica Ulivelli, Maria Felice Ghilardi, Nicola De Stefano, Simone Rossi

**Affiliations:** Department of Molecular, Cellular and Biomedical Sciences, City University of NewYork, School of Medicine, New York, NY 10031, United States; Department of Medicine, Surgery and Neuroscience, Siena Brain Investigation and Neuromodulation Lab (Si-BIN Lab), Unit of Neurology and Clinical Neurophysiology, University of Siena, Siena 53100, Italy; Department of Molecular, Cellular and Biomedical Sciences, City University of NewYork, School of Medicine, New York, NY 10031, United States; Department of Medicine, Surgery and Neuroscience, Siena Brain Investigation and Neuromodulation Lab (Si-BIN Lab), Unit of Neurology and Clinical Neurophysiology, University of Siena, Siena 53100, Italy; Department of Medicine, Surgery and Neuroscience, University of Siena, Siena 53100, Italy; Department of Molecular, Cellular and Biomedical Sciences, City University of NewYork, School of Medicine, New York, NY 10031, United States; Department of Molecular, Cellular and Biomedical Sciences, City University of NewYork, School of Medicine, New York, NY 10031, United States; Department of Molecular, Cellular and Biomedical Sciences, City University of NewYork, School of Medicine, New York, NY 10031, United States; Department of Medicine, Surgery and Neuroscience, University of Siena, Siena 53100, Italy; Siena Imaging SRL, Siena 53100, Italy; Department of Physiotherapy, Medicine and Biomedical Sciences, NEUROcom (Neuroscience and Motor Control Group), Universidade da Coruña, Biomedical Institute of A Coruña (INIBIC), and Galician Brain Stimulation Center, A Coruña 15006, Spain; Department of Mental Health, The First Affiliated Hospital, Guangxi Medical University, Nanning 530021, China; Department of Medicine, Surgery and Neuroscience, University of Siena, Siena 53100, Italy; Department of Medicine, Surgery and Neuroscience, Siena Brain Investigation and Neuromodulation Lab (Si-BIN Lab), Unit of Neurology and Clinical Neurophysiology, University of Siena, Siena 53100, Italy; Oto-Neuro-Tech Conjoined Lab, Policlinico Le Scotte, University of Siena, Siena 53100, Italy; Department of Medicine, Surgery and Neuroscience, University of Siena, Siena 53100, Italy; Department of Information Engineering and Mathematics, University of Siena, Siena 53100, Italy; Department of Information Engineering and Mathematics, University of Siena, Siena 53100, Italy; Department of Medicine, Surgery and Neuroscience, University of Siena, Siena 53100, Italy; Siena Imaging SRL, Siena 53100, Italy; Department of Medicine, Surgery and Neuroscience, University of Siena, Siena 53100, Italy; Department of Medicine, Surgery and Neuroscience, University of Siena, Siena 53100, Italy; Department of Medicine, Surgery and Neuroscience, Siena Brain Investigation and Neuromodulation Lab (Si-BIN Lab), Unit of Neurology and Clinical Neurophysiology, University of Siena, Siena 53100, Italy; Department of Medicine, Surgery and Neuroscience, Siena Brain Investigation and Neuromodulation Lab (Si-BIN Lab), Unit of Neurology and Clinical Neurophysiology, University of Siena, Siena 53100, Italy; Department of Medicine, Surgery and Neuroscience, Siena Brain Investigation and Neuromodulation Lab (Si-BIN Lab), Unit of Neurology and Clinical Neurophysiology, University of Siena, Siena 53100, Italy; Department of Medicine, Surgery and Neuroscience, Siena Brain Investigation and Neuromodulation Lab (Si-BIN Lab), Unit of Neurology and Clinical Neurophysiology, University of Siena, Siena 53100, Italy; Goldsmiths, University of London, London SE14 6NW, UK; Department of Medicine, Surgery and Neuroscience, University of Siena, Siena 53100, Italy; Department of Molecular, Cellular and Biomedical Sciences, City University of NewYork, School of Medicine, New York, NY 10031, United States; Department of Medicine, Surgery and Neuroscience, University of Siena, Siena 53100, Italy; Department of Medicine, Surgery and Neuroscience, Siena Brain Investigation and Neuromodulation Lab (Si-BIN Lab), Unit of Neurology and Clinical Neurophysiology, University of Siena, Siena 53100, Italy; Oto-Neuro-Tech Conjoined Lab, Policlinico Le Scotte, University of Siena, Siena 53100, Italy

**Keywords:** fatigue, EEG, beta oscillations, multiple sclerosis, beta ERD/ERS

## Abstract

Fatigue is one of the most disabling symptoms of multiple sclerosis (MS), yet its neurobiology remains unclear, and there are no objective biomarkers. Previous studies using electroencephalography alone have revealed altered movement-related beta Event-Related Desynchronization (ERD) and Synchronization (ERS) dynamics in fatigued patients, but without providing mechanistic insight. In this cross-sectional study, we combined electroencephalography with transcranial magnetic stimulation, structural magnetic resonance imaging with diffusion tensor imaging (DTI), and clinical measures to probe the mechanistic basis of movement-related beta modulation depth (ERS-ERD) and its link with fatigue. Based on the Fatigue Severity Scale score (FSS), we enrolled 41 relapsing-remitting MS patients, 19 with clinically significant fatigue, 22 without (aged 25–55 years, 25 females), alongside 18 age- and sex-matched healthy volunteers. Participants underwent neuropsychological assessment, blood sampling for inflammatory and neurodegeneration-related biomarkers, structural magnetic resonance imaging with DTI to assess grey and white matter integrity, transcranial magnetic stimulation to quantify cortical excitatory and inhibitory balance, and continuous electroencephalography during 300 cued pinch movements to characterize movement-related beta dynamics. Compared with non-fatigued patients and healthy volunteers, fatigued patients exhibited reduced beta peak ERD to ERS modulation (*P* < 0.001), especially in frontal regions. The modulation depth correlated with fatigue severity (*ρ* = −0.54, *P* = 0.0006), intracortical facilitation (*ρ* = 0.49, *P* = 0.0009), and caudate nucleus volume (*ρ* = 0.35, *P* = 0.010). A nested elastic-net logistic regression integrating demographic, clinical, structural, and functional markers showed robust held-out performance (mean Receiver Operating Characteristic-Area Under the Curve = 0.92, Precision Recall-Area Under the Curve = 0.83, accuracy = 0.89, Brier score = 0.10). Variables with the strongest protective association with fatigue were higher intracortical facilitation, better mental health, larger caudate volume, greater beta modulation over the frontal regions, and higher corticospinal tract and superior longitudinal fasciculus integrity. These findings support frontal beta modulation as a mechanistically grounded, non-invasive biomarker of central fatigue in multiple sclerosis and highlight its potential utility for clinical diagnosis and targeted therapeutic intervention.

## Teaser

Tatti, Benelli *et al*. showed that patients with multiple sclerosis and fatigue display reduced movement-related beta modulation compared with healthy and non-fatigued individuals. Reduced beta modulation correlated with greater fatigue, lower glutamatergic activity and reduced structural integrity, independently of depression, thus supporting beta modulation as a biomarker of fatigue.

## Graphical Abstract

**Graphical Abstract:**
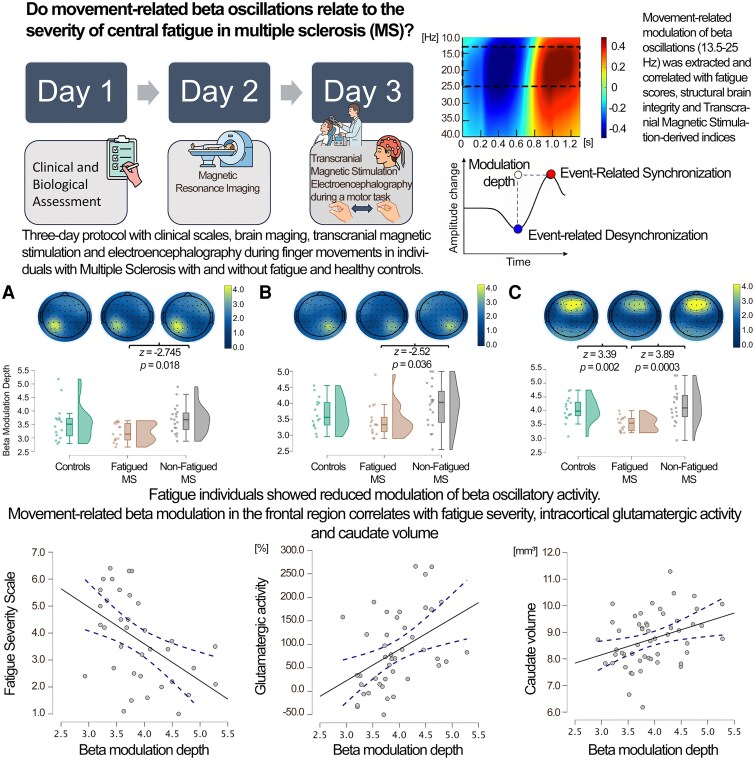

## Introduction

Fatigue is a prevalent and debilitating symptom of multiple sclerosis (MS), affecting up to 78% of patients.^1,2^ Although described as a single entity, fatigue can manifest in several forms, including physical (e.g. muscle weakness), cognitive (e.g. ‘brain fog’), and psychosocial (e.g. feeling overwhelmed) fatigue, making it difficult to reach a consensus on its definition. Further, its subjective nature and variability across individuals pose a significant challenge in terms of diagnosis and the development of interventions to address it. While the mechanisms underlying fatigue remain poorly understood,^3^ several pathophysiological contributors have been proposed, including: (i) structural damage, such as white matter (WM) demyelination and grey matter (GM) atrophy,^4^ (ii) neuroinflammation, which alters brain metabolism, neurotransmitter balance, and neural excitability, and (iii) maladaptive functional recruitment.^5-9^ Among these mechanisms, the maladaptive recruitment hypothesis suggests that MS fatigue may arise from the engagement of additional or non-optimal brain areas to compensate for structural damage, ultimately leading to inefficient network activity and increased subjective effort. Recent studies highlighted the role of frontal and subcortical circuits in regulating effort and motor control, linking their dysfunction to fatigue in neurological conditions.^10-13^ For example, the cortico-striato-thalamo-cortical network, a crucial hub for the selection, modulation, and reinforcement of motor, cognitive, and emotional actions, has been consistently implicated in the pathogenesis of fatigue across different disorders,^14,15^ with thalamic dysfunction emerging as a core node in the ‘fatigue network’.^15-17^ Increasing evidence also points to the superior longitudinal fasciculus (SLF), a major WM tract supporting motor planning, sensorimotor integration, attention, and executive control.^18^ Although it lacks direct projections to primary motor areas, the SLF connects cortical regions that integrate sensory input with action planning, thereby supporting movement modulation through attentional and executive mechanisms. Reduced SLF integrity has been linked to poorer fine motor skills in ADHD^19^ and slower post-stroke reaction times in reaching tasks,^20^ underscoring its relevance for higher-order motor function. More recently, researchers have emphasized the role of metacognition and interoception, proposing that altered interpretation of internal bodily states may lead to an exaggerated perception of effort and energy depletion.^21-24^ Accordingly, fatigue emerges from disrupted sensory attenuation mechanisms, leading to an inaccurate assessment of the effort/energetic costs of the motor output.^25^ Given the role of beta oscillations (13.5–25 Hz) in sensorimotor integration and movement preparation, abnormalities in movement-related Event-Related Desynchronization (ERD) and Synchronization (ERS) in the beta range may thus serve as a possible neurophysiological marker of fatigue.

Beta oscillations are generally associated with inhibitory control, mediated by local GABAergic activity,^26-28^ decreased cortical excitability,^29-31^ and the maintenance of internal motor and cognitive states.^32-34^ Beta ERD, which begins with movement preparation, reflects motor disinhibition and sensory attenuation, and is tied to movement features. The post-movement beta ERS, while weakly correlated with kinematics,^35-40^ is thought to reflect network re-inhibition and the integration of afferent feedback to refine motor predictions.^32,41^ Its amplitude correlates with reduced motor uncertainty and increases with learning and practice, returning to baseline after rest.^42-47^

A recent hypothesis based on the analysis of disorders characterized by psychomotor slowing and fatigue, such as major depression, Parkinson's disease, and MS,^48^ links impaired beta ERD/ERS to reduced energy availability and regulation.^49^ Supporting this, beta oscillations have been linked to key metabolic and neurotransmitter systems, including dopaminergic and GABAergic signaling, and lactate levels.^50,51^ Further, exercise-induced fatigue is accompanied by increased beta activity in frontal and sensorimotor regions,^52,53^ together with reduced striatal dopamine D2 receptor expression and dopamine release,^54^ suggesting that beta rhythm alterations may reflect disrupted metabolic and dopaminergic homeostasis. As recently proposed,^55,56^ these mechanisms may underlie central fatigue in MS.

Building upon these insights, our study extends seminal findings^57^ of reduced fronto-central beta ERD and ERS in patients with MS (pwMS) with fatigue. Here, we used a multimodal approach to characterize the relationship between MS fatigue, movement-related beta oscillations, structural and functional indices derived from Magnetic Resonance Imaging (MRI), and Transcranial Magnetic Stimulation (TMS). Specifically, we compared fatigued pwMS (MS-F), non-fatigued pwMS (MS-NF), and healthy controls (HC) during simple movements to determine whether alterations in beta oscillatory dynamics are associated with neurophysiological and structural differences, as well as with clinical measures.

## Materials and methods

### Participants

Forty-one patients with relapsing-remitting multiple sclerosis (RRMS) according to the McDonald criteria,^58^ and 18 age-matched healthy controls (mean age: 41.61 ± 11.07 years, 11 women) were recruited from the Neurology Unit of the University Hospital ‘Santa Maria alle Scotte’ in Siena, Italy. All patients were right-handed, as determined by Oldfield's Handedness Inventory.^59^ Exclusion criteria included: (i) any contraindication to TMS, such as epilepsy, a cardiac pacemaker, or metal implants, (ii) an MS relapse in the previous three months, (iii) upper limb paresis, (iv) onset of novel neurological symptoms within four weeks of the data collection. Major secondary causes of fatigue, including anaemia, thyroid dysfunction, medication effects, and sleep disorders, were clinically screened and excluded.

Based on a cut-off score of 4 on the Fatigue Severity Scale (FSS),^60^ patients with MS (pwMS) were classified as Fatigued [MS-F: *N* = 19, 14 female; age: 41.8 ± 10.8 SD, FSS: 5.24 ± 0.82 SD, EDSS: 1.763 ± 0.61 (0–2.5); disease duration: 107.7 ± 90.7 months] and Non-Fatigued [MS-NF: 22, 11 female age: 42.4 ± 9.6 years; FSS: 2.39 ± 0.83 SD; EDSS: 1.48 ± 0.48 (1–2.5); disease duration: 107.5 ± 91.2 months]. All participants maintained stable pharmacological regimens in the weeks preceding data collection (Supplementary Table 1).

This investigation, performed following the ethical principles of the Declaration of Helsinki and its subsequent amendments, was approved by the local Ethics Committee (Brainsight 2025–28). All participants gave informed consent to each experimental procedure.

### Experimental design

**Figure 1:**
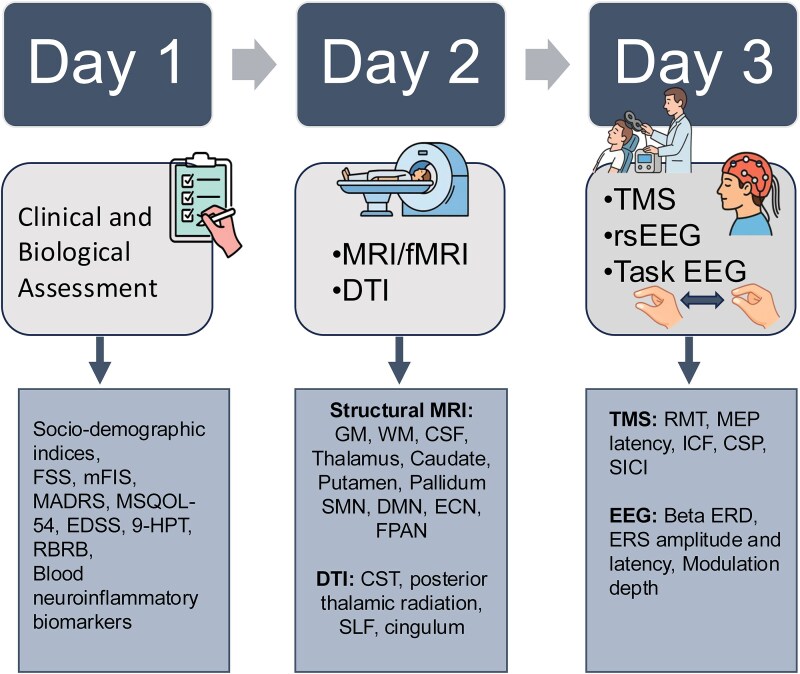
**Overview of the experimental protocol.** Day 1: clinical, demographic, and blood-based biomarker assessments. Day 2: MRI, functional MRI (fMRI), and DTI scans for structural and connectivity analysis. Day 3: TMS and electroencephalography (EEG) to assess cortical excitability and beta (13.5–25 Hz) oscillatory activity. The boxes below outline specific measures included in this study. 9-HPT, Nine-Hole Peg Test; CSF, cerebrospinal fluid; CSP, cortical silent period; CST, corticospinal tract; DMN, default mode network; DTI, diffusion tensor imaging; ECN, executive control network; EDSS, Expanded Disability Status Scale; EEG, electroencephalography; ERD, event-related desynchronization; ERS, event-related synchronization; fMRI, functional magnetic resonance imaging; FPAN, frontoparietal attention network; FSS, Fatigue Severity Scale; GM, grey matter; ICF, intracortical facilitation; MADRS, Montgomery-Åsberg Depression Rating Scale; MEP, motor evoked potential; mFIS, modified Fatigue Impact Scale; MRI, magnetic resonance imaging; MSQOL-54, Multiple Sclerosis Quality of Life-54; RBRB, Repeatable Battery for the Assessment of Neuropsychological Status; RMT, resting motor threshold; rsEEG, resting-state electroencephalography; SICI, short-interval intracortical inhibition; SLF, superior longitudinal fasciculus; SMN, sensorimotor network; TMS, transcranial magnetic stimulation; WM, white matter.

The study followed a three-day protocol to assess behavioural, neuroimaging, and neurophysiological domains (Fig. 1). First, participants completed a 1-h session including sociodemographic and clinical assessments to evaluate fatigue, disability, psychological status, and both sensorimotor and cognitive functioning. Further, blood samples were collected to assess peripheral immune activation. MRI scans (e.g. FLAIR, T1-weighted, DTI) were acquired on day two to assess structural and functional brain integrity. Lastly, participants completed TMS testing to assess cortical excitation-inhibition balance, followed by EEG recordings at rest and during motor and working memory tasks. Blood inflammatory and neurodegeneration-related biomarkers and MRI data were acquired and analyzed with standard clinical protocols. Acquisition parameters, procedures, and analyses are described in the Supplementary Materials.

### Sociodemographic and clinical data collection

Participants provided sociodemographic information, including age, sex, years of education, medication history, Body Mass Index (BMI), and Basal Metabolic Rate (BMR). Depression severity was assessed with the Montgomery-Åsberg Depression Rating Scale (MADRS),^61^ while manual dexterity and motor function were evaluated with the 9-Hole Peg Test (9-HPT),^62^ both for the dominant (Right) and non-dominant (Left) hands. Cognitive performance was evaluated using the Rao Brief Repeatable Battery,^63^ which assesses verbal and visuospatial memory, information processing speed, attention, and verbal fluency (Supplementary Materials). PwMS further completed the Modified Fatigue Impact Scale (mFIS)^64^ to evaluate the impact of fatigue on physical (Ph), cognitive (Cog), and psychosocial functioning (Psy), the Multiple Sclerosis Quality of Life-54 (MSQOL-54)^65^ to assess quality of life, and the Expanded Disability Status Scale (EDSS)^66^ to determine disability level.

### Motor task data collection

EEG activity was recorded at least 2 hours after the TMS session, while participants performed 300 cued pinch movements. Seated comfortably in front of a computer screen with their right arm resting on a stable surface, participants responded to a green circle that appeared at random intervals between 1.3 and 1.5 s. Upon each cue, they were instructed to pinch a force sensor between their thumb and index finger with maximum strength, then release immediately. Due to a sensor malfunction, force data were excluded from analysis.

### Electromyographic (EMG) data collection and analysis

EMG activity from the first dorsal interosseus (FDI) muscle was recorded throughout the motor task. The signal was first segmented into 1.5 s epochs based on the green circle onset and rectified as:


EMG_rect(t)=|EMG(t)−mean(EMG)

To extract the smoothed envelope of the rectified signal, a second-order Butterworth low-pass filter was applied using the *filtfilt* function. The filter cutoff frequency of 20 Hz was adjusted by 25% to ensure an effective −3 dB frequency at the desired cutoff. The filtered EMG signal was normalized by dividing it by the maximum value. EMG signals were visually inspected to mark the onset and offset of muscular activity. Trials with excessive noise, with no clear EMG peak, were excluded (Mean: 286.13 ± 14.27 SD kept movements). EMG activity onset time and contraction duration were extracted for statistical analysis.

### EEG data collection and analysis

EEG recordings were acquired using a 64-channel cap with a G-Tech amplifier system (g.®HIamp) (sampling rate: 256 Hz; reference electrode: nasion, Nz). Electrode placement followed the international 10–20 system, with two additional EOG electrodes positioned vertically above and below the left eye and two electrodes on the left and right mastoids. Electrode impedances were maintained below 10 kΩ throughout the recording. All recorded data were preprocessed at CUNY, School of Medicine, using a custom EEGLAB-based pipeline on MATLAB (v.2023a).^67^ The continuous EEG signal was filtered with a Finite Impulse Response (FIR) filter in the 1–90 Hz range, and notch filtered (46–54 Hz) to eliminate line noise. Filter order was automatically determined based on the sampling rate and transition bandwidths. The signal was then visually inspected to identify and remove channels with poor signal quality. Independent Component Analysis (ICA, Infomax algorithm) was performed to detect artefacts such as eye movements, high-frequency muscle activity, heartbeat, and other periodic EEG artefacts. The IC Label toolbox, which assigns a percentage-based classification of independent components into categories,^68^ was used to reject components classified as Muscle (30–100%), Eye (20–100%), Heart (80–100%), or Line Noise (50–100%) (Mean kept components: 29.8 ± 12.15 SD). The signal was then segmented into 2-s epochs (−0.5–1.5 s) centred on the appearance of the green circle on the display (300 epochs). Electrodes with poor signal quality were reconstructed using spherical spline interpolation, and external channels were excluded, resulting in 61 average-referenced electrodes. All subsequent EEG analyses were performed using the MATLAB-based Fieldtrip Toolbox.^69^

### Movement-related beta ERD and ERS analysis

The preprocessed data were transformed by applying the current source density algorithm (*ft_scalpcurrentdensity*), which enhances the spatial resolution of scalp EEG and reduces the influence of broadly distributed volume-conducted signals.^70^ Time-frequency representations were then computed by convolving the signal with Complex Morlet Wavelets (1–90 Hz, 0.5 Hz increments) over the entire time window (10 ms steps, 3 to 10 cycles).

To extract movement-related beta (13.5–25 Hz) ERD and ERS peak values, the data were baseline-corrected by subtracting and dividing the average signal of the entire trial time window (−0.5–1.5 s). A data-driven approach was employed to identify the Region of Interest (ROI) showing the maximal ERD and ERS activity. For each participant, the beta ERS-ERD peak-to-peak difference (i.e. beta modulation depth) was computed to identify the electrode with the highest modulation depth and its six nearest neighbours; to guide this search, the peak ERD and ERS were first determined within three broad regions corresponding to the frontal (AFz, AF3, AF4, Fz, F1, F2, F3, F4, F5, F6, FCz, FC1, FC2, FC3, FC4), left sensorimotor (CZ, C2, C4, C6, CP6, CP4, CP2, CPz, P6, P4, P2, Pz), and right sensorimotor (Cz, C1, C3, C5, CPz, CP1, CP3, CP5, Pz, P1, P3, P5) areas. Peak ERD was defined as the minimum value of beta power between the onset of the green circle (0 s) to 0.7 s after; ERS magnitude was extracted as the maximum value in the interval between 0.7 and 1 s. Based on the resulting topography, for each participant, the electrode with the highest activity and its six neighbours were selected for each of three ROIs: frontal, left, or right sensorimotor areas (Frontal, Left, and Right ROIs). After the ROIs selection, peak beta ERD and beta ERS magnitude and timing, along with the beta modulation depth, were re-computed within each personalized ROI and extracted for each trial and participant. The average beta activity was also extracted to evaluate baseline differences across groups.

### Statistical analysis

Normality and homogeneity of variance were assessed using the Shapiro-Wilk test and Levene's test. Because several variables violated these assumptions, non-parametric tests were applied to all group comparisons for consistency. Group differences for all continuous metrics were assessed using Kruskal–Wallis tests (JASP v 0.19.3), followed by Dunn's post-hoc tests with Bonferroni correction for multiple comparisons (two-tailed tests, *α* = 0.05). Bayesian ANOVAs using default JASP priors were also conducted to support inferential statistics results. Chi-square tests were used to assess sex distribution across groups.

Given significant group differences in depression and dexterity levels, mediation analyses were run to examine whether such variables explained the relationship between the examined indices. The indirect (IE), direct (DE), and total effects (TE) were estimated and 95% bias-corrected percentile bootstrap confidence intervals (CI) (1000 bootstrap resamples) were computed for each effect.

Spearman correlation analyses assessed the relationships between beta activity and other variables displaying significant group differences, with 95% bootstrap CI (1000 resamples) to estimate correlation strength. To control for multiple comparisons, we applied a Bonferroni correction across the three primary EEG measures (ERD, ERS, and modulation depth), which set a significance threshold of *α* = 0.0167.

Given the confounding effect of depression on fatigue and beta activity, multiple linear regression was also performed to test whether beta modulation predicted fatigue (FSS scores) independently of depressive symptoms (MADRS). Separate models were run for the Frontal, Left, and Right ROIs beta modulation. Standardized *β* coefficients, *F* values, and *P*-values were reported.

Finally, to identify the best predictors of fatigue, we modelled a binary outcome (0 = MS-F, 1 = MS-NF) using a pre-specified set of variables, including demographics/clinical data (age, sex, disease duration, education, MADRS, MSQOL-54 physical and mental health, 9-HPT right), MRI volumetry (WM, caudate, thalamus; lesion count and volume), TMS indices (CSP, SICI, ICF, RMT, MEP latency), beta modulation (Left, Right, Frontal ROIs), and diffusion FA (CST left/right, SLF left/right). Participants with missing data on any of these predictors were excluded (*N* = 28 of 41 participants).

We used penalized logistic regression with an elastic-net penalty using the MATLAB function *lassoglm*. Hyperparameters were tuned by stratified 5-fold nested cross-validation (CV): in each outer split, ∼80% of the data served as the training set and ∼20% as the held-out test set. Within the training set, a 5-fold inner CV searched across *α* values (0.1–1.0, LASSO = 1) and ∼100 log-spaced *λ* values. The *α* and *λ* pair with the lowest inner-CV deviance was refit on the full training set and then evaluated on the held-out test set. Predictions from all outer test sets were combined to compute ROC-AUC, PR-AUC, accuracy, sensitivity, specificity, balanced accuracy, and the Brier score. To obtain stable estimates and selection frequencies, we repeated the full nested CV procedure 50 times with new splits and reported mean performance with percentile 95% intervals.

For model interpretability, we ran a descriptive penalized logistic regression using predictors selected in ≥50% of outer splits, applying a small L2 ridge penalty to avoid separation. Continuous predictors were z-scored, and sex was entered as a categorical variable. From this refit, we report regression coefficients (*β*), odds ratios (ORs) with 95% stratified bootstrap CI, and pseudo-*R*2 values (McFadden, Cox–Snell, Nagelkerke).

## Results

### Clinical and demographic differences across groups

The three groups had comparable gender, age, BMI, and BMR. HC showed higher education levels than MS-F (Mean difference HC versus MS-F: 3.13 years ± 1.05 SE) (Table 1). Kruskal–Wallis tests on motor functioning showed a significant effect for the right 9-HPT, with poorer performance of the MS-F group compared with HC (*P* = 0.01). Differences for the left hand were less prominent (*P* = 0.052). MADRS scores were greater in MS-F individuals compared with the HC (*P* = 0.0002) and MS-NF (*P* = 0.003) (Supplementary Table 2). Cognitive status (Supplementary Table 3) and blood-derived inflammation- and neurodegeneration-related biomarkers values (Supplementary Table 4) were comparable across groups.

**Table 1:** Descriptives and group comparisons of demographic and clinical measures

	MS_F	MS_NF	HC	*Test*	*P*	BF_10_
Female (%)	13 (68%)	11 (50%)	11 (61%)	1.47	0.48	0.24
Age	41.84 ± 10.56	42.41 ± 9.62	41.61 ± 11.07	0.03	0.984	0.14
Education	13.26 ± 3.53	14.14 ± 3.06	16.39 ± 3.01	9.32	**0.009**	3.83
BMI	25.18 ± 5.80	25.38 ± 5.29	22.21 ± 2.86	3.80	0.149	0.83
BMR	1453.97 ± 229.66	1539.30 ± 174.82	1485.76 ± 206.61	1.84	0.398	0.27
Right 9-HPT	20.66 ± 3.01	23.94 ± 3.58	21.66 ± 3.57	9.11	**0.011**	**3.15**
Left 9-HPT	22.12 ± 4.85	24.06 ± 2.22	24.20 ± 7.50	5.92	0.052	0.25
MADRS	8.56 ± 4.26	4.68 ± 5.38	2.67 ± 2.35	17.93	**0.002**	60.67

Descriptives and group comparisons of demographic and clinical measures for the Fatigued MS (MS-F) and Non-Fatigued MS (MS-NF), and Healthy Controls (HC) groups. Data are presented as mean ± SD or percentage. Test statistics include: Χ^2^ = Chi-square test for gender differences; H = Kruskal–Wallis H statistic; *P* = *P*-value; BF₁₀ = Bayes Factor indicating the strength of evidence in favour of the alternative hypothesis over the null. Significant group differences (*P* < 0.05) are shown in bold. Abbreviations: BMI, body mass index; BMR, basal metabolic rate; 9-HPT, Nine-Hole Peg Test; MADRS, Montgomery-Äsberg Depression Rating Scale.

Clinical data analyses between MS-F and MS-NF groups (Table 2) revealed similar disease duration but significant differences in FSS scale, mFIS total score, and its subdomains, with the MS-F group reporting higher fatigue scores across all dimensions. The MS-F group also displayed statistically higher EDSS scores compared with MS-NF (*P* = 0.033), a result that, however, is not supported by Bayesian analysis (BF_10_ = 0.935) and has minimal clinical relevance. MSQOL-54 scores, reflecting health-related quality of life, were comparable for physical health but the MS-F group showed significantly lower scores for mental health (Table 2).

**Table 2:** Descriptives and group comparisons of clinical and quality-of-life measures

	MS-F	MS-NF	*H*	*P*	BF_10_
Disease duration (months)	107.69 ± 90.71	107.53 ± 90.94	0.006	0.937	0.31
EDSS	1.76 ± 0.61	1.48 ± 0.47	4.54	**0.033**	0.935
FSS	5.24 ± 0.82	2.40 ± 0.86	29.90	**< 0.0001**	1.364 × 10 ^+ 10^
mFIS Tot	42.74 ± 11.43	22.05 ± 14.41	16.66	**< 0.0001**	1462.64
mFIS Cog	18.05 ± 6.20	9.96 ± 7.54	11.05	**0.0009**	45.94
mFIS Ph	20.84 ± 5.53	10.77 ± 7.10	16.05	**< 0.0001**	1345.17
mFIS Psy	3.63 ± 2.06)	1.32 ± 1.287	13.70	**0.0002**	241.82
MSQOL54 Ph	4.68 ± 5.38	5.74 ± 2.04	2.09	0.149	0.64
MSQOL54 Mh	4.22 ± 1.50	5.62 ± 1.91	5.395	**0.020**	3.18

Descriptives and group comparisons of clinical and quality-of-life measures between Fatigued (MS-F) and Non-Fatigued (MS-NF) MS groups. Data are presented as mean ± SD or percentage. Test statistics include: H = Kruskal–Wallis H statistic; *P* = *P*-value; BF₁₀ = Bayes Factor indicating the strength of evidence in favour of the alternative hypothesis over the null. Significant group differences (*P* < 0.05) are shown in bold. Abbreviations: EDSS, Expanded Disability Status Scale; FSS, Fatigue Severity Scale; mFIS, Modified Fatigue Impact Scale; Tot, total score; Cog, cognitive subscale; Ph, physical subscale; Psy, psychosocial subscale; MSQOL54, Multiple Sclerosis Quality of Life-54; Mh, mental health composite.

### Brain volume alterations identified by structural MRI

Both MS groups had a greater number and volume of WM lesions compared with HC (Number of lesions, *H* = 29.05, *P* < 0.0001, HC versus MS-F: *z* = −4.71, *P* < 0.0001, HC versus MS-NF: −32.57, *z* = −4.73, *P* < 0.0001; Lesions volume, *H* = 35.64, *P* < 0.0001, HC versus MS-F: *z* = −5.51, *P* < 0.0001, HC versus MS-NF: *z* = −4.87, *P* < 0.0001). No differences were observed between MS-F and MS-NF groups. Volumetry analyses showed significant differences in the total brain volume (*H* = 6.894, *P* = 0.03), with lower values in MS-F compared with HC (HC versus MS-F: *z* = 2.611, *P* = 0.01). While GM differences were not significant (*H* = 3.11, *P* = 0.21), MS-F had lower WM volume compared with HC (*H* = 7.013; *P* = 0.03; Mean diff: 22.35 ± 8.25 SE, *z* = 2.504, *P* = 0.02; MS-F and MS-NF, Mean diff: −14.53 ± 7.86 SE, *z* = −2.02, *P* = 0.07). Bayesian post-hoc tests confirmed strong evidence for a difference between HC and MS-F and anecdotal evidence for the MS-F and MS-NF comparison (Supplementary Table 5).

Group differences were also found for the thalamus (*H* = 16.18, *P* = 0.0003) and caudate nucleus volumes (*H* = 6.357, *P* = 0.04), with reduced thalamic volume in both MS groups compared with HC (HC versus MS-F, Mean diff: 2.01 ± 0.504 SE, *z* = 3.66, *P* = 0.0004; HC versus MS-NF, Mean diff: 1.62 ± 0.49, *z* = 3.36, *P* = 0.001), and caudate volume in MS-F compared with HC (HC versus MS-F: Mean diff: 0.83 ± 0.31 SE, *z* = 2.52, *P* = 0.036). Mediation analyses showed that indirect effects via depression (MADRS) or motor performance (9-HPT) was not significant, indicating that these variables did not mediate the observed volume reductions (Supplementary Table 6).

### Fractional anisotropy alterations in major WM tracts

Analysis of Fractional anisotropy (FA) values revealed significant microstructural differences. In the right CST, we found group differences (*H* = 7.02, *P* = 0.03, BF_10_ = 6.96), with reduced FA in the MS-F group compared with HC (*z* = 2.54, *P* = 0.033, BF_10_ = 41.32). A different pattern emerged in the left CST (*H* = 20.23, *P* < 0.0001; BF_10_ = 1265.79), with the MS-NF displaying higher FA values than the other two groups (HC and MS-NF: *z* = −3.99, *P* < 0.0001, BF_10_ = 173.99; MS-F and MS-NF: *z* = −3.669, *P* = 0.0004, BF_10_ = 78.66). Similarly, the right posterior thalamic radiation exhibited significant alterations (*H* = 12.153, *P* = 0.002, BF_10_ = 13.15) with greater FA in MS-NF compared with HC (*z* = −2.836, *P* = 0.007, BF_10_ = 13.46), and MS-F (*z* = −3.099, *P* = 0.003; BF_10_ = 4.99). No significant differences were observed for the left thalamic radiation (*H* = 4.49, *P* = 0.106, BF_10_ = 0.63). Analysis of the right SLF showed significant differences (*H* = 17.55, *P* = 0.00015, BF_10_ = 294.82) with higher FA for the MS-NF compared with HC (*z* = −3.445, *P* = 0.00086, BF_10_ = 22.24) and MS-F (*z* = −3.69, *P* = 0.00033; BF_10_ = 156.53). A similar pattern was observed in the left SLF (*H* = 10.77, *P* = 0.005; BF_10_ = 11.23), with higher FA in MS-NF patients than HC (*z* = −3.27, *P* = 0.002, BF_10_ = 44.36). No significant group differences were noted in the cingulum on either side (Right cingulum: *H* = 4.467, *P* = 0.107, BF_10_ = 0.44; Left cingulum: *H* = 2.462, *P* = 0.292, BF_10_ = 0.34). Depression level and motor dexterity do not mediate the observed effects (Supplementary Table 7).

### TMS-derived markers of GABAergic and glutamatergic activity

The three groups did not differ in terms of Resting Motor Threshold (RMT) (*H* = 1.64, *P* = 0.44), Motor Evoked Potential (MEP) latency (*H* = 3.48, *P* = 0.18), SICI (*H* = 3.80, *P* = 0.15), and Cortical Silent Period (CSP) (*H* = 4.92, *P* = 0.09). We found significant group differences for ICF (*H* = 21.45, *P* = 0.00002, BF_10_ = 8532.64), with the MS-F patients displaying significantly reduced facilitation compared with both HC (Mean Diff.: 67.15 ± 22.20 SE, *z* = 2.70, *P* = 0.021, BF_10_ = 22.99) and MS-NF groups (Mean Diff.: −125.76 ± 21.10, *z* = −4.59, *P* = 0.00001, BF_10_ = 3067.34). Mediation analysis confirms no mediation effect of MADRS and 9-HPT (Dummy coded: MS-F = 1, HC = 0, MADRS, DE: Estimate = −59.27, *P* = 0.017; IE: Estimate = −7.60, *P* = 0.64; TE: −66.87, *P* = 0.0003; Right 9-HPT, DE: Estimate = −83.25, *P* = 0.00001; IE: Estimate = 15.27, *P* = 0.12; TE: Estimate = −67.99, *P* = 0.0002).

### EMG activity during motor performance and movement-Related Beta ERD and ERS Modulation

Analyses on EMG activity during the motor task revealed significant group differences for reaction time (RT, *H* = 10.63, *P* = 0.005, BF₁₀ = 8.109), with HC exhibiting faster RTs compared with the MS-F group (Table 3). No differences were observed for the duration of the muscular contraction (*H* = 4.54, *P* = 0.103, BF₁₀ = 0.37).

**Table 3:** Dunn's post hoc EMG onset and latency comparisons across groups

	Comparison	*z*	*P*	BF_10_
EMG Onset	HC versus MS-F	−3.19	**0.004**	9.686
	HC versus MS-NF	−2.14	0.097	2.209
	MS-F versus MS-NF	1.13	0.775	0.796
EMG duration	HC versus MS-F	−2.11	0.104	0.888
	HC versus MS-NF	−1.26	0.622	0.397
	MS-F versus MS-NF	0.90	1.00	0.429

Dunn's *post hoc z* and Bonferroni-corrected *P*-values, Bayesian prior and posterior odds, and Bayes Factors (BF₁₀) for between-group comparisons of EMG onset and latency. Significant group differences (*P* < 0.05) are shown in bold. Abbreviations: EMG, electromyography; MS-F, multiple sclerosis with fatigue; MS-NF, multiple sclerosis without fatigue; HC, healthy controls; BF₁₀, Bayes factor in favor of the alternative hypothesis.

**Figure 2:**
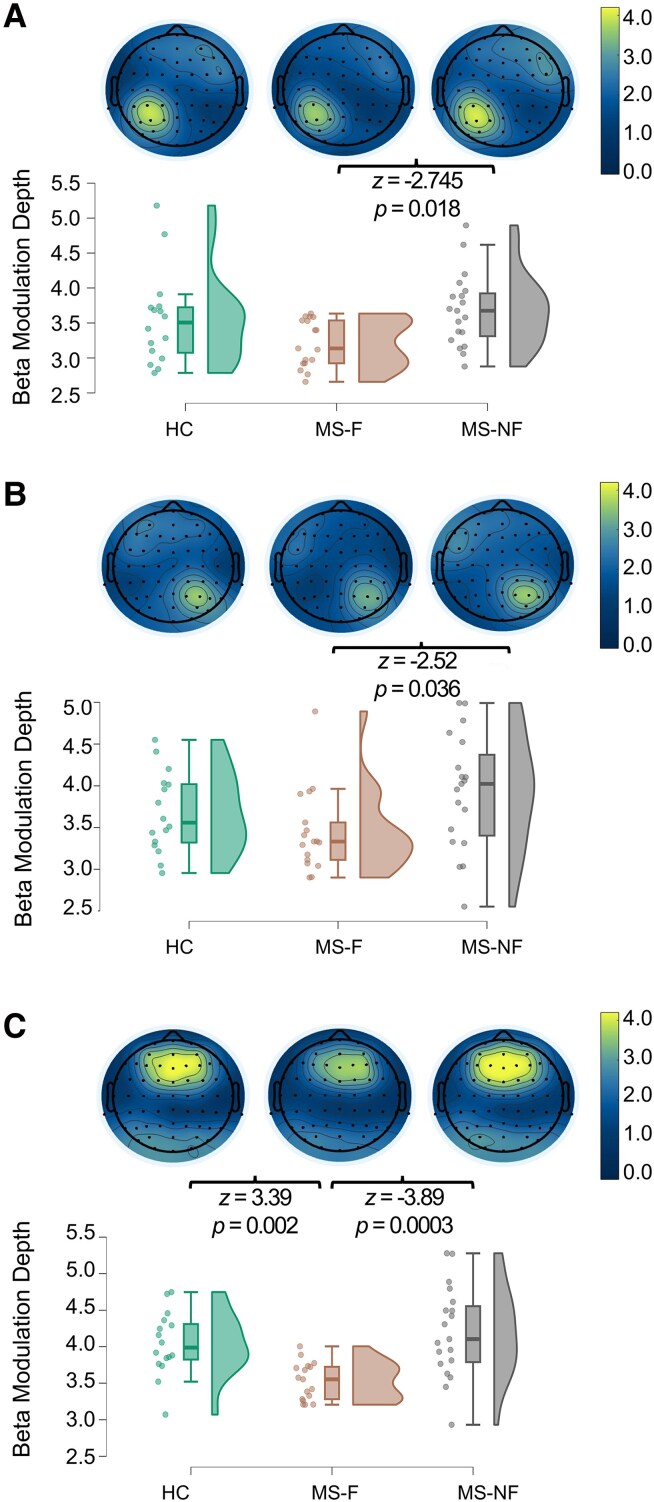
**Group comparisons of beta modulation depth and scalp topography.** Topographical plots displaying scalp distribution (Top) and raincloud plots of beta modulation depth (peak ERS-ERD) computed within subject-specific Regions of Interest (ROIs) over the Left Top panel (**A**), Right Middle panel (**B**), and Frontal Bottom panel (**C**) ROIs for healthy controls (HC, Left ROI: *n* = 17, Right ROI: *n* = 16, Frontal ROI: *n* = 17), fatigued (MS-F, *n* = 17 for all ROIs), and non-fatigued MS (MS-NF, *n* = 19 for all ROIs) patients with MS. Each data point represents the ROI-specific beta modulation depth value for an individual participant. Group differences were assessed using Kruskal–Wallis tests followed by Dunn's post-hoc tests with Bonferroni correction; *z* and *P*-values for significant post-hoc comparisons are reported above the raincloud plots. Colour bars and raincloud plot *y*-axes represent movement-related beta modulation depth, computed as Event-Related Synchronization (ERS) minus ERD in the beta band and normalized to the mean beta power in the movement window (unitless).

All three groups exhibited distinct movement-related beta ERD and ERS dynamics in the Right, Left, and Frontal ROIs (Fig. 2, Supplementary Fig. 1). However, in a few participants, distinct beta dynamics could not be identified during the expected task intervals (i.e. no visible beta ERD and ERS activity could be observed). To minimize variability and ensure consistency in ROI-based analyses, these participants were excluded. Thus, subsequent analyses were conducted on 16 MS-F, 19 MS-NF, and 17 HC participants.

Kruskal–Wallis tests on beta ERD revealed group differences in the Frontal and Right ROIs (Table 4). MS-F patients showed reduced beta ERD in the Frontal ROI compared with both MS-NF (*z* = 3.83, *P* = 0.0004) and HC (*z* = 2.48, *P* = 0.04), and in the Right ROI compared with MS-NF (*z* = 2.904, *P* = 0.01). No group differences emerged for the Left ROI. Comparisons between MS-NF and HC were not significant (Left ROI: *z* = 0.313, *P* = 1; Right ROI: *z* = 0.51, *P* = 1; Frontal ROI: *z* = 1.29, *P* = 0.59). MS-F displayed reduced beta ERS in all three ROIs compared with MS-NF (Left: *z* = −2.786, *P* = 0.016; Right: *z* = −2.481, *P* = 0.039; Frontal: *z* = −3.889, *P* = 0.0003), and in the Frontal ROI compared with HC (*z* = 3.420, *P* = 0.002). These findings were corroborated by Bayesian statistics, with strong evidence especially for the Frontal ROI (Supplementary Table 8).

**Table 4:** Beta ERD/ERS metrics and mean Beta power across groups by ROI

		Peak Beta power change					
		MS-F	MS-NF	HC	*H*	*P*	BF_10_
**ERD**	**Left**	−0.83 (0.032)	−0.83 (0.032)	−0.85 (0.03)	5.86	0.053	1.71
	**Right**	−0.83 (0.03)	−0.86 (0.04)	−0.86 (0.03)	9.34	**0.009**	5.53
	**Frontal**	−0.85 (0.02)	−0.89 (0.03)	−0.88 (0.02)	15.05	**0.0005**	186.67
**ERS**	**Left**	2.38 (0.32)	2.83 (0.50)	2.79 (0.73)	8.13	**0.017**	1.98
	**Right**	2.62 (0.47)	3.07 (0.66)	2.82 (0.46)	6.16	**0.046**	1.29
	**Frontal**	2.68 (0.24)	3.31 (0.60)	3.20 (0.43)	17.87	**0.0001**	101.94
**Beta modulation depth**	**Left**	3.21 (0.34)	3.69 (0.52)	3.65 (0.75)	8.01	**0.018**	2.18
	**Right**	3.45 (0.50)	3.93 (0.69)	3.68 (0.48)	6.35	**0.042**	5.53
	**Frontal**	3.53 (0.26)	4.20 (0.62)	4.07 (0.44)	17.74	**0.0001**	129.41

		Peak latency (s)					
		MS-F	MS-NF	HC	*H*	*P*	BF_10_
**ERD**	**Left**	0.41 (0.04)	0.41 (0.03)	0.41 (0.03)	0.55	0.76	0.15
	**Right**	0.40 (0.04)	0.41 (0.03)	0.41 (0.03)	0.06	0.97	0.16
	**Frontal**	0.41 (0.04)	0.41 (0.02)	0.41 (0.03)	1.47	0.48	0.15
**ERS**	**Left**	1.05 (0.05)	1.06 (0.04)	1.04 (0.05)	0.66	0.72	0.21
	**Right**	1.06 (0.04)	1.06 (0.04)	1.06 (0.06)	0.18	0.91	0.15
	**Frontal**	1.05 (0.04)	1.06 (0.03)	1.05 (0.05)	0.38	0.83	0.17

		Average beta power					
		MS-F	MS-NF	HC	*H*	*P*	BF_10_
**Average Beta**	**Left**	67.42 (47.36)	76.72 (51.96)	107.09 (101.16)	1.80	0.41	0.42
	**Right**	67.18 (43.23)	75.58 (49.99)	118.99 (125.38)	1.61	0.45	0.62
	**Frontal**	62.63 (43.21)	69.96 (40.36)	71.69 (54.88)	1.38	0.50	0.17
	**All channels**	70.68 (52.09)	81.27 (49.04)	99.64 (98.97)	1.24	0.54	0.25

Mean and standard deviation (Mean ± SD) of peak power change from baseline (dimensionless), latency (in seconds) for beta ERD, ERS, and modulation depth, and average beta activity across three groups (MS-F, MS-NF, and HC) in the Left, Right, and Frontal ROIs. *H* and *P* values refer to Kruskal–Wallis test results. *BF₁₀* indicates Bayes Factor in favor of the alternative hypothesis from Bayesian ANOVAs. Significant *P*-values (*P* < 0.05) are shown in bold. Abbreviations: MS-F, multiple sclerosis with fatigue; MS-NF, multiple sclerosis without fatigue; HC, healthy controls; ROI, region of interest; ERD, event-related desynchronization; ERS, event-related synchronization.

There were no group differences in the latency of peak ERD and ERS and in the average beta signal over the selected ROIs and all the scalp electrodes, suggesting that these findings were specific to movement-related oscillatory dynamics (Table 4). Mediation analyses ruled out any confounding effects of MADRS and 9-HPT scores (Supplementary Table 9).

### Beta modulation is linked to structural MS abnormalities and cortical glutamatergic facilitation

Negative correlations were found between WM volume and ERD in the three ROIs: Left (*ρ* = −0.28, *P* = 0.046), Right (*ρ* = −0.32, *P* = 0.019), and Frontal ROIs (*ρ* = −0.33, *P* = 0.017), with the latter surviving Bonferroni correction (*α* = 0.0167). Positive relationships were observed between caudate volume and both ERS (Left ROI: *ρ* = 0.33, *P* = 0.016; Frontal ROI: *ρ* = 0.36, *P* = 0.008) and modulation depth (Left ROI: *ρ* = 0.33, *P* = 0.015; Frontal ROI: *ρ* = 0.35, *P* = 0.010).

Right SLF FA correlated negatively with ERD over the Frontal ROI (*ρ* = −0.31, *P* = 0.026) and positively with ERS over both Frontal (*ρ* = 0.37, *P* = 0.008) and Right ROIs (*ρ* = 0.30, *P* = 0.028). Again, only the association with Frontal ERS remained significant after correction. Similar patterns were also observed with modulation depth (Frontal ROI: *ρ* = 0.31, *P* = 0.023; Right ROI: *ρ* = 0.36, *P* = 0.009), with the Right ROI effect surviving correction. No significant correlations were observed between thalamus volume, CST FA, thalamic radiation FA, and beta indices. Correlation analyses between movement-related beta modulation and TMS-derived measures showed that ICF was negatively correlated with beta ERD in all three ROIs: Left (*ρ* = −0.40, *P* = 0.008), Right (*ρ* = −0.47, *P* = 0.002), and Frontal (*ρ* = −0.49, *P* = 0.0008) and survived Bonferroni correction. Positive correlations between ICF and ERS were also observed for the Left (*ρ* = 0.38, *P* = 0.011) and Frontal ROIs (*ρ* = 0.49, *P* = 0.0008), both significant even after correction, but not for the Right ROI (*ρ* = 0.30, *P* = 0.053). Similar results were found between ICF and modulation depth, with significant positive associations at the Left (*ρ* = 0.38, *P* = 0.012) and Frontal ROIs (*ρ* = 0.49, *P* = 0.0009), both surviving Bonferroni correction, while the Right ROI effect did not (*ρ* = 0.30, *P* = 0.051).

### Movement-related beta modulation is associated with fatigue severity

**Figure 3:**
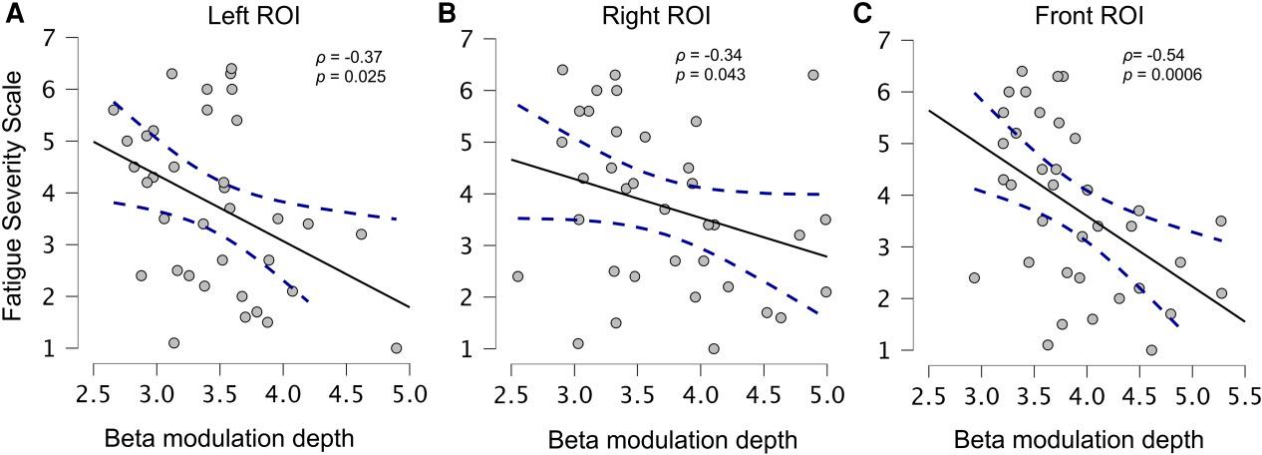
**Relationship between fatigue and beta modulation depth.** Scatterplots showing Spearman correlations between FSS scores and beta-modulation depth (computed as Event-Related Synchronization, ERS, minus Event-Related Desynchronization, ERD, in the beta band and normalized to the mean beta power in the movement window; unitless) in Left (Panel **A**, *n* = 36), Right (Panel **B**, *n* = 36), and Frontal (Panel **C**, *n* = 36) ROIs. Solid lines indicate least-squares regression fits, and shaded bands represent 95% CI. Each dot represents an individual MS participant who completed the FSS. Spearman's *ρ* and uncorrected *P*-values are shown in the upper-right of each panel. The Bonferroni-corrected significance threshold for these three comparisons is *α* = 0.017.

Spearman correlation analyses revealed that FSS showed mild positive correlations with beta ERD over the Left (*ρ* = 0.35, *P* = 0.034), Right (*ρ* = 0.34, *P* = 0.044), and Frontal ROIs (*ρ* = 0.42, *P* = 0.011). After Bonferroni correction, only the correlation in the Frontal ROI remained significant. Conversely, negative correlations with FSS were found for beta ERS over the Left (*ρ* = −0.38, *P* = 0.023), Right (*ρ* = −0.35, *P* = 0.035), and Frontal ROIs (*ρ* = −0.56, *P* = 0.0004). As for beta ERS, FSS was negatively associated with modulation depth across the Left (*ρ* = −0.37, *P* = 0.025), Right (*ρ* = −0.34, *P* = 0.043), and Frontal ROIs (*ρ* = −0.54, *P* = 0.0006). After correction, only the Frontal ROI effect remained significant (Supplementary Fig. 2A, B, Fig. 3).

Further significant relationships were observed between beta ERS, especially in the Frontal ROI, and the mFIS Total (Supplementary Fig. 2D), physical and cognitive subscales scores (Frontal ROI: mFIS TOT: *ρ* = −0.45, *P* = 0.006, mFIS physical: *ρ* = −0.43, *P* = 0.009, mFIS cognitive: *ρ* = −0.40, *P* = 0.017). A significant negative correlation also emerged between Frontal beta ERD and the mental health subscale of the MSQOL-54 (*ρ* = −0.44, *P* = 0.009). No notable correlations were observed between Right and Left 9-HPT scores, MADRS, and beta indices (Supplementary Table 10).

To determine whether beta modulation depth predicts fatigue independently of depressive symptoms, a multiple regression was conducted with FSS as the dependent variable and beta modulation depth and MADRS scores as predictors. Results indicated that, for the Frontal and Left ROIs, both higher depressive symptom burden and lower beta modulation depth were significant predictors of fatigue (Frontal ROI model: *F*_(2,32)_ = 9.73, *P* = 0.0005; Left ROI model: *F*_(2,32)_ = 8.82, *P* = 0.0009).

Higher MADRS scores (*β* = 0.40, *t* = 2.825, *P* = 0.008) and lower beta modulation depth (*β* = −0.38, *t* = −2.686, *P* = 0.011) were each associated with greater fatigue severity in the model testing the Frontal ROI. A similar result was obtained for the Left ROI model (MADRS: *β* = 0.430, *t* = 2.985, *P* = 0.005, Beta modulation: *β* = −0.348, *t* = −2.416, *P* = 0.022). Differently, beta modulation in the Right ROI did not emerge as a significant predictor of fatigue (*F*_(2,32)_ = 6.84, *P* = 0.003; Beta modulation: *β* = −0.25, *t* = −1.677, *P* = 0.103; MADRS: *β* = 0.473, *t* = 3.191, *P* = 0.003), reinforcing the idea that the link between fatigue and beta activity is specific for the frontal and the dominant sensorimotor network.

### Multimodal markers predict fatigue status in pwMS

To identify stable predictors of fatigue in MS, we finally fit a penalized logistic regression model to a multimodal set of demographic, clinical, structural, and functional measures.

The model (*N* = 28) showed solid held-out performance with good discrimination ability (ROC-AUC = 0.887), precision-recall performance (PR-AUC = 0.828), and accuracy of 0.857. Repetition of the cross-validation 50 times yielded similar results [mean ROC-AUC = 0.922, (95% CI: 0.838–0.982), PR-AUC 0.830, (95% CI: 0.742–0.904), accuracy = 0.894, balanced accuracy = 0.703, Brier score = 0.100], indicating good discrimination.

Stability analysis shows a coherent predictor set; features with mean selection frequency (MSF) > 0.50 were: ICF (MSF = 1.00), Left CST FA (MSF = 1.00), sex (MSF = 0.996), Right SLF FA (MSF = 0.988), MSQOL-54_MH (MSF = 0.936), caudate volume (MSF = 0.792), frontal beta modulation (MSF = 0.788), Right CST FA (MSF = 0.628), disease duration (MSF = 0.596), Left SLF FA (MSF = 0.588), and MEP latency (MSF = 0.504). Variables with MSF < 0.50 were excluded from interpretation.

For interpretability, we refit a penalized logistic model on the selected variables (MS-F = 1, MS-NF = 0). Several measures showed a protective association with fatigue: male sex (OR = 0.82, 95% CI: 0.76–0.94), better mental health (MSQOL-54: OR = 0.74, 95% CI: 0.65–0.87), larger caudate volume (OR = 0.82, 95% CI: 0.70–0.96), higher ICF (OR = 0.71, 95% CI: 0.62–0.86), greater frontal beta modulation (OR = 0.74, 95% CI: 0.65–0.87), and higher white-matter integrity in the Right CST (OR = 0.79, 95% CI: 0.69–0.91), Left CST (OR = 0.68, 95% CI: 0.61–0.76), and Right SLF (OR = 0.71, 95% CI: 0.62–0.84).

Disease duration (OR = 0.88, 95% CI: 0.73–1.09), MEP latency (OR = 0.88, 95% CI: 0.75–1.08), and Left SLF FA (OR = 0.98, 95% CI: 0.82–1.18) were not clearly associated, as their CI crossed 1 (Supplementary Table 1^1^). Despite the small sample size, Pseudo-*R*^2^ values indicated good model fit (McFadden = 0.592, Cox–Snell = 0.559, Nagelkerke = 0.746), consistent with strong separation between fatigued and non-fatigued patients.

## Discussion

Our study shows reduced movement-related beta modulation in fatigued pwMS, particularly in the frontal regions. Notably, these alterations are not general features of MS pathology, but rather distinguish fatigued from non-fatigued patients, underscoring the specific link between beta activity and fatigue. Indeed, altered movement-related beta modulation was not attributable to differences in depression or motor impairment and was associated with fatigue levels, lower glutamatergic activity, caudate volume, and WM integrity.

Our results on beta modulation align with and extend previous EEG and MEG research, highlighting altered beta ERD and ERS in pwMS.^57,71-73^ The observed reduced frontal beta modulation in fatigued pwMS compared with HC closely mirrors previous findings^57^ of diminished fronto-central beta ERD and ERS in fatigued pwMS, with these reductions correlating with fatigue severity. Similarly, another study^72^ found decreased ERS in the left fronto-central region in pwMS compared with HC, although their study did not differentiate between fatigued and non-fatigued patients and included different MS subtypes. Further, MEG studies reported reduced beta ERS in the pre- and post-central gyri in pwMS during both isometric knee extension^71^ and cued finger button press,^73^ which was interpreted as evidence of faulty internal models in pwMS. Evidence indicates movement-related beta modulation as arising from the interplay between sensory and motor regions.^33,34,41,74^ As resting beta oscillations maintain the motor network in an idling state, beta ERD reflects the release of this inhibition and the rise in corticospinal excitability to initiate and execute movement. Importantly, beta ERD occurs even in the absence of overt movement, such as during motor imagery, and is thought to reflect the suppression of sensory reafference that facilitates efficient motor output. Conversely, beta ERS is believed to reflect the re-stabilization of the sensorimotor network and the integration of sensory feedback for assessing and updating the executed motor representations.^32,75^ Reduced ERS amplitude has been observed with the mismatch between the intended action and its sensory consequences,^76^ suggesting that a blunted ERS could support the destabilization of current motor predictions to update them using incoming sensory input.

This evaluative process is thought to rely on a distributed network that includes the medial prefrontal cortex, supplementary motor areas, and anterior cingulate cortex, all regions critically involved in prediction error monitoring, action planning, and effort allocation. In healthy brains, top-down predictions and internal models (both motor and cognitive) reduce the reliance on sensory processing. However, when such resources are limited, the brain shifts to bottom-up processing, which is less efficient and metabolically costly.^25^

Thus, the reduced frontal beta modulation in fatigued pwMS may reflect disrupted top-down control and sensory attenuation, increasing processing load and perceived effort. This interpretation aligns with recent models of fatigue that implicate impaired sensory attenuation as a core mechanism underlying the experience of central fatigue in MS.^25,77^

Given this general regulatory function; beta modulation can also be interpreted in terms of available energetic resources to sustain predictive processing and efficient use of sensory input.^49^ Thus, we speculate that the blunted beta ERD/ERS may index a system operating near its metabolic limits, leading to altered inhibitory/excitation balance.

Support for this interpretation comes from the observed reduced resting ICF, which indexes *N*-methyl-D-aspartate (NMDA) receptor-mediated glutamatergic neurotransmission, in fatigued patients. Notably, ICF was negatively correlated with beta ERD and positively with beta ERS and emerged as one of the most stable predictors of fatigue.

While resting beta activity has been predominantly associated with GABAergic inhibition,^28,78,79^ its modulation during movement depends on a dynamic balance between cortical excitation and inhibition.^74^ Indeed, GABA reuptake inhibition increases resting beta power and reduces movement-related ERD, suggesting reduced recruitment of cortical pyramidal neurons during movement.^28,80,81^ In contrast, both sensorimotor training and neuromodulation techniques that increase cortical excitability consistently amplify beta ERS over frontal and sensorimotor areas.^42,44-47,82,83^ Supporting this view, EEG-fMRI studies demonstrate increased post-central gyrus activation during beta ERS, indicating that this phase reflects active processing of sensory reafferences, potentially mediated by glutamatergic signaling.^84^

While previous investigations reported changes in cortical excitability,^11,85-89^ the relationship between glutamatergic signaling and fatigue remains unclear. Some studies^11,87^ found no significant differences in ICF between fatigued and non-fatigued pwMS, although one reported reduced MEP amplitude following a facilitatory 5 Hz rTMS protocol in the fatigued group.^87^ Conversely, another investigation demonstrated a significant reduction in ICF in fatigued individuals.^86^ Importantly, disease-modifying interventions for MS, such as high-dose corticosteroids^85^ and 3,4-diaminopyridine,^90,91^ have been shown to increase ICF in pwMS.

In our study, fatigued pwMS also showed non-significant trends toward reduced cortical inhibition, as reflected by shorter CSP, a previously described finding that is reversed by amantadine as an anti-fatigue drug,^92^ and higher SICI. However, these differences did not reach statistical significance, likely due to the limited sample size.

Taken together, our findings suggest that movement-related beta modulation is shaped not only by inhibitory GABAergic activity but also, if not more, by the integrity of excitatory cortical glutamatergic pathways. The observed association between reduced ICF and beta modulation further suggests that altered beta dynamics might reflect short-term plasticity mechanisms or fluctuating metabolic support for sensorimotor function.

Our findings also provide insights into the intricate relationship between depression and fatigue.^93^ While fatigued pwMS showed significant mild depressive symptoms, our findings indicate that the relationship between beta modulation and fatigue is not merely driven by depression burden, thus suggesting distinct neurophysiological mechanisms. While depressive symptoms may amplify fatigue through motivational and affective pathways, reduced beta modulation likely reflects alterations within networks supporting sensorimotor control and perceived effort.

Another interesting finding concerns the relationship between WM microstructure and beta modulation. While fatigued pwMS displayed reduced FA in the CST, posterior thalamic radiation, and SLF, non-fatigued pwMS showed higher FA values, particularly in the SLF and left CST. These observations align with previous evidence linking fatigue to alterations in key WM structures, notably the fronto-striatal and fronto-frontal networks, SLF, CST, and posterior thalamic radiation.^94-98^ Importantly, we also identified significant correlations between SLF FA, previously linked to motor regulation and post-stroke motor recovery,^99^ and beta modulation. Consistently, our elastic-net model identified both CST and SLF WM integrity as stable protective factors against fatigue, thereby raising the possibility that preserved structure may support compensatory mechanisms.

Together, these results suggest that, in fatigued patients, impaired WM integrity disrupts structural-functional coupling, reducing cortical efficiency and increasing perceived effort. Therapies aimed at enhancing WM integrity, such as clemastine fumarate, a first-generation antihistamine,^100^ and high-dose biotin,^101^ have been proven promising in supporting myelin repair and axonal function. In addition, motor rehabilitation and non-invasive brain stimulation techniques (e.g. transcranial electrical and magnetic stimulation^102-104^) and tailored exercise protocols^105^ have been shown to enhance structural plasticity. These approaches pave the way for possible strategies to reduce MS-related fatigue.^106^

## Limitations and future directions

This study has several limitations. First, the small sample size limits generalizability and statistical power, and replication in larger cohorts is needed to confirm these findings. Second, although we quantified overall lesion load, its spatial distribution was not mapped. Future studies should include lesion-symptom mapping to clarify how lesion location contributes to excitability, beta activity, and fatigue. Third, our focus on WM lesion burden overlooked cortical demyelination, which likely contributes to abnormal oscillatory activity. While the measures of GM volume we included in our study may indirectly reflect cortical demyelination through its contribution to atrophy, they do not allow its specific quantification. Future studies with dedicated MRI sequences, such as Double Inversion Recovery, should investigate cortical demyelination directly. Fourth, our DTI protocol omitted spinal cord imaging. Given the contribution of spinal pathology to motor function and TMS-derived excitability measures, the absence of spinal cord data may obscure structure-function relationships. Further, we did not assess cortico-thalamic tracts, which are key to the regulation of brain oscillations and fatigue; future studies should include them to clarify their role and link to fatigue and neurophysiological measures.

Finally, limiting the sample to RRMS reduced variability but limits generalizability to progressive forms, and the cross-sectional design precludes causal inference. Longitudinal studies are needed to track how beta activity, WM, excitability, and fatigue unfold over time.

## Conclusions

Our findings demonstrate that fatigue in RRMS is associated with reduced movement-related beta modulation, impaired glutamatergic facilitation, and decreased WM integrity, independent of lesion burden. These alterations converge to identify movement-related beta oscillations as a promising non-invasive biomarker of central fatigue in MS, reflecting underlying neurophysiological and structural changes. Objective markers such as these may guide future strategies to assess and address MS fatigue.

## Supplementary Material

fcag043_Supplementary_Data

## Data Availability

Due to privacy and consent restrictions, the individual-level EEG and clinical datasets are not publicly available but can be shared in anonymized form upon request to the corresponding authors and subject to institutional approvals. The MATLAB scripts used for data analysis in this study are deposited in a public GitHub repository (https://github.com/ElisaT-Neuro/MS_beta_fatigue).
