# Supplementary material for: Linking movement-related beta oscillations to cortical excitability, structural damage, and fatigue in multiple sclerosis
Source: Brain Commun. 2026 Mar 12;8(2):fcag043. doi: 10.1093/braincomms/fcag043 (PMC12980577; doi:10.1093/braincomms/fcag043)
Supplement: fcag043_Supplementary_Data [file fcag043_supplementary_data.docx]

**Supplementary Materials**

**Blood biomarkers assessment**

Because fatigue in Multiple Sclerosis (MS) may involve inflammatory and neurodegenerative processes, we extracted blood biomarkers to capture peripheral signatures of neuroinflammation and neural damage. Markers such as serum neurofilament light chain (NfL) and glial fibrillary acidic protein (GFAP) provide objective indices of neuroaxonal and astroglial injury, while cytokine profiling offers insight into systemic immune dysregulation. To extract such indices, fasting blood samples were collected between 8:00 and 10:00 AM. Serum was separated by centrifugation, aliquoted into sterile polypropylene tubes, and stored at -80 °C until analysis. NfL and GFAP levels were quantified using the Simoa™ Neurology 2-Plex B kit (Quanterix, Billerica, MA, USA) on the SR-X platform. Samples were diluted 1:4 and assayed in randomized order on 96-well plates. Quality controls fell within expected ranges, and inter-plate coefficients of variation were below 10%. Analyses were conducted blind to group identity, and verified concentrations (pg/mL) were used for group comparisons. A subset of samples was analyzed for cytokines using a multiplex bead-based flow cytometry assay (LEGENDplex™ Human Essential Immune Response Panel, BioLegend, San Diego, CA, USA). The panel included Interleukin-1 beta (IL-1β), interleukin-2 (IL-2), interleukin-4 (IL-4), interleukin-6 (IL-6), interleukin-8 (IL-8), interleukin-10 (IL-10), interleukin-12 p70 (IL-12p70), interleukin-17A (IL-17A), tumor necrosis factor alpha (TNF-α), interferon gamma (IFN-γ), transforming growth factor beta-1 (TGF-β1), C-X-C motif chemokine ligand 10 (CXCL10, also known as interferon gamma–induced protein 10 [IP-10]), C-C motif chemokine ligand 2 (CCL2, also known as monocyte chemoattractant protein-1 [MCP-1]). Data were acquired on a FACSCanto II cytometer and processed with LEGENDplex™ v8.0 software. Samples were diluted 1:2 and run in randomized order after standard calibration. Cytokine concentrations (pg/mL) were exported to the database for statistical analysis. Results of group comparisons obtained with the Kruskal-Wallis test and Bayesian ANOVAs are reported in Supplementary Table 4.

**Cognitive assessment**

A cognitive assessment was conducted using the Rao Brief Repeatable Battery (RBRB)^1^, which evaluates cognitive domains commonly affected in MS. The battery includes measures of verbal learning and memory (Selective Reminding Test), visuospatial memory (Spatial Recall Test), processing speed and attention (Paced Auditory Serial Addition Test; Symbol Digit Modalities Test), and verbal fluency (Word List Generation). No significant differences were found between the three groups (Supplementary Table 3)**.**

**Magnetic Resonance Imaging (MRI) data acquisition**

MRI measures were included to investigate the structural changes in our sample that could explain fatigue and their relationship with beta modulation. MRI data were collected at Meyer University Hospital in Florence, Italy, using a 3T Philips scanner (Philips Medical Systems, Best, The Netherlands) equipped with an eight-channel head coil. Image acquisition was guided by a sagittal scout to identify the anterior and posterior commissures, with all sequences acquired in the axial plane aligned to the bi-commissural line. Proton density (PD) and T2-weighted (T2W) images were obtained using a dual-echo turbo spin-echo sequence (TR/TE1/TE2 = 4000/10/100 ms; voxel size = 1×1×3 mm^3^). T1 weighted (T1) image was obtained using a gradient echo sequence (TR/TE = 10/3.9 ms; voxel size = 1x1x1 mm3). Diffusion tensor imaging (DTI) was obtained using a spine echo sequence (TR/TE = 6000/84; voxel size = 2.4 x 2.4 x 2.5 mm3; b-values = 900; number of directions = 32). All scans underwent visual inspection for quality assurance and were processed centrally. Brain tissue segmentation masks for gray matter (GM) and white matter (WM) were generated using a custom preprocessing pipeline built on FSL tools (<https://fsl.fmrib.ox.ac.uk>). Voxel-wise analyses of grey matter (GM) and white matter (WM) volumes were conducted following normalization to standard space and registration to study-specific templates. Importantly, although we also collected functional MRI (fMRI) data, they were not included in this study.

**MRI Brain Volumetry analyses**

T1-weighted (T1-W) images were used for brain volumetry after lesion filling to avoid bias in GM estimation ^2^. Brain segmentation was performed using SIENAX2 ^3,4^, an updated version of the SIENAX tool within the FMRIB Software Library (FSL) that provides skull-size–normalized volumetric estimates ^5,6^. SIENAX2 performs brain extraction, registration to standard space, and tissue segmentation into GM, WM, and cerebrospinal fluid (CSF) using FAST ⁶. Deep grey matter structures, including the thalamus, caudate nucleus, putamen, globus pallidus, nucleus accumbens, hippocampus, and amygdala, were segmented using the FSL-FIRST module integrated within the SIENAX2 pipeline ^7^.

All regional and total brain volumes were normalized for head size by multiplying by the SIENAX2 volumetric scaling factor (VSCALING). Cortical GM was parcellated using the Desikan–Killiany atlas ^8^, and lobe volumes (frontal, temporal, parietal, occipital) were extracted from predefined MNI-space masks. Functional network masks, including the sensorimotor and default-mode networks, were non-linearly registered to individual T1-W images. Cortical thickness was computed using FreeSurfer.

For this study, we focused on total brain, GM, WM, CSF, thalamus, caudate, putamen, and pallidum, as well as the sensorimotor (SMN) and default-mode (DMN) networks. A detailed account of the full pipeline is provided in our companion paper Benelli, Tatti et al. (under review).

**WM Lesion Detection and Analysis**

WM lesions were segmented semi-automatically from FLAIR/T2-hyperintense images using a custom deep-learning tool to generate lesion masks. Each segmentation was reviewed by an expert (R.C.) and manually corrected when necessary, using FSLview (FMRIB Software Library, FSL). Cortical lesions were identified using Double Inversion Recovery (DIR) imaging. Binarized lesion masks were registered to the MNI152 standard space using linear registration (FLIRT) ^6^ followed by nonlinear registration (FNIRT) via FSL.

**Diffusion Tensor Imaging (DTI) analysis**

DTI preprocessing was performed using FSL’s eddy tool, which corrects for eddy current distortions, subject motion, and signal dropout. The FMRIB Diffusion Toolbox (FDT) was used to compute Fractional Anisotropy (FA) maps by fitting a diffusion tensor model to each voxel. Brain extraction was performed using BET ^4^. All FA and Mean Diffusivity (MD) images were registered to the FMRIB58 standard-space template ^9^. Tract-Based Spatial Statistics (TBSS) ^10^ was used for voxel-wise analysis of WMM microstructure. For region-based analysis, mean FA and MD values were extracted using *fslstats* within WM tracts defined by the JHU-ICBM 1mm atlas (<https://neurovault.org/collections/264/>). To explore the relationship between WM integrity and beta oscillatory activity, FA values were correlated with EEG-derived beta ERD and ERS across fatigued and non-fatigued MS participants. MD values were not included in this study. Given the relevance of fatigue to both motor and cognitive domains, and the role of corticospinal, thalamic, and associative tracts in movement-related beta ERD and ERS, here we focused on the corticospinal tract (CST), superior longitudinal fasciculus (SLF), thalamic radiations, and cingulum bundle. A complete account of the DTI analyses is reported in our companion paper Benelli, Tatti et al. (under review).

**Transcranial Magnetic Stimulation (TMS)**

TMS measures were included to evaluate cortical excitability and inhibitory–excitatory balance, which have been shown to be altered in MS fatigue. By probing glutamatergic and GABAergic circuits, TMS provides direct insights into motor cortex function and synaptic plasticity, thus allowing us to assess the link between such alterations, fatigue, and beta modulation.

Neuronavigated-TMS was conducted following international safety guidelines ^11^. Structural T1-weighted MRI scans were acquired for each participant and co-registered with a stereotaxic neuronavigation system (NDI, Waterloo, Canada) using BrainNET software (EBNeuro Ltd, Florence, Italy). The T1 image and reconstructed head model were aligned in the Polaris infrared-optical tracking system (Nortern Digital Inc., ON, Canada) using anatomical landmarks (nasion, vertex, preauricular points). TMS was delivered over the left primary motor cortex (M1) hand area using a figure-eight coil (70 mm) connected to an ATES magnetic stimulator (EBNeuro Ltd). The motor hotspot for the right First Dorsal Interosseous (FDI) muscle was identified with the coil positioned at 45° to the midline. Surface electromyographic (EMG) electrodes recorded Motor Evoked Potentials (MEPs) using the NExT Station system. Resting motor threshold (RMT) was defined as the minimum intensity required to produce 50% of liminal responses (∼50 μV) in 10 consecutive trials (Rossini et al. 2015), while the muscle was at rest ^12^. MEP onset was marked by the amplitude exceeding ±2 SD of the EMG baseline, with latency measured from the TMS pulse to MEP onset. ^12^ Cortical Silent Period (CSP) was measured by delivering 15 pulses at 120% RMT during a 20% maximal voluntary contraction pinch grip. CSP duration was defined from MEP onset to the return of pre-TMS EMG activity and averaged across 10–15 trials ^12^ and was averaged across 10–15 traces per participant.

Paired-pulse TMS was also used to assess intracortical inhibition and facilitation mechanisms. Short Intra-Cortical Inhibition (SICI), mediated by GABA-A receptors ^13^, and intracortical facilitation (ICF), an index of glutamatergic activity ^14^, were assessed using a subthreshold conditioning stimulus set to 80% of RMT followed by a test stimulus at 120% of RMT. A 3-ms interstimulus interval (ISI) was used for SICI and a 10-ms ISI for ICF, with 15 stimuli delivered randomly at intervals of 5–8 seconds ^15–18^. The amplitude of the conditioned MEPs was expressed as a percentage of the unconditioned response, with reductions indicating inhibition (SICI) and increases indicating facilitation (ICF).

**References**

1. Bever CT, Grattan L, Panitch HS, Johnson KP. The Brief Repeatable Battery of Neuropsychological Tests for Multiple Sclerosis: a preliminary serial study. *Mult Scler*. 1995;1(3):165-169. doi:10.1177/135245859500100306

2. Battaglini M, Jenkinson M, De Stefano N. Evaluating and reducing the impact of white matter lesions on brain volume measurements. *Human Brain Mapping*. 2012;33(9):2062-2071. doi:10.1002/hbm.21344

3. Smith SM, Jenkinson M, Woolrich MW, et al. Advances in functional and structural MR image analysis and implementation as FSL. *NeuroImage*. 2004;23:S208-S219. doi:10.1016/j.neuroimage.2004.07.051

4. Smith SM, Zhang Y, Jenkinson M, et al. Accurate, robust, and automated longitudinal and cross-sectional brain change analysis. *Neuroimage*. 2002;17(1):479-489. doi:10.1006/nimg.2002.1040

5. Jenkinson M, Bannister P, Brady M, Smith S. Improved optimization for the robust and accurate linear registration and motion correction of brain images. *Neuroimage*. 2002;17(2):825-841. doi:10.1016/s1053-8119(02)91132-8

6. Jenkinson M, Smith S. A global optimisation method for robust affine registration of brain images. *Med Image Anal*. 2001;5(2):143-156. doi:10.1016/s1361-8415(01)00036-6

7. Patenaude B, Smith SM, Kennedy DN, Jenkinson M. A Bayesian model of shape and appearance for subcortical brain segmentation. *NeuroImage*. 2011;56(3):907-922. doi:10.1016/j.neuroimage.2011.02.046

8. Desikan RS, Ségonne F, Fischl B, et al. An automated labeling system for subdividing the human cerebral cortex on MRI scans into gyral based regions of interest. *Neuroimage*. 2006;31(3):968-980. doi:10.1016/j.neuroimage.2006.01.021

9. Rueckert D, Sonoda LI, Hayes C, Hill DLG, Leach MO, Hawkes DJ. Nonrigid registration using free-form deformations: application to breast MR images. *IEEE Transactions on Medical Imaging*. 1999;18(8):712-721. doi:10.1109/42.796284

10. Smith SM, Jenkinson M, Johansen-Berg H, et al. Tract-based spatial statistics: Voxelwise analysis of multi-subject diffusion data. *NeuroImage*. 2006;31(4):1487-1505. doi:10.1016/j.neuroimage.2006.02.024

11. Rossi S, Antal A, Bestmann S, et al. Safety and recommendations for TMS use in healthy subjects and patient populations, with updates on training, ethical and regulatory issues: Expert Guidelines. *Clinical Neurophysiology*. 2021;132(1):269-306. doi:10.1016/j.clinph.2020.10.003

12. Rossini PM, Burke D, Chen R, et al. Non-invasive electrical and magnetic stimulation of the brain, spinal cord, roots and peripheral nerves: Basic principles and procedures for routine clinical and research application. An updated report from an I.F.C.N. Committee. *Clinical Neurophysiology*. 2015;126(6):1071-1107. doi:10.1016/j.clinph.2015.02.001

13. Di Lazzaro V, Pilato F, Dileone M, et al. Segregating two inhibitory circuits in human motor cortex at the level of GABAA receptor subtypes: A TMS study. *Clinical Neurophysiology*. 2007;118(10):2207-2214. doi:10.1016/j.clinph.2007.07.005

14. Liepert J, Mingers D, Heesen C, Bäumer T, Weiller C. Motor cortex excitability and fatigue in multiple sclerosis: a transcranial magnetic stimulation study. *Mult Scler*. 2005;11(3):316-321. doi:10.1191/1352458505ms1163oa

15. Nakamura H, Kitagawa H, Kawaguchi Y, Tsuji H. Intracortical facilitation and inhibition after transcranial magnetic stimulation in conscious humans. *J Physiol*. 1997;498 ( Pt 3)(Pt 3):817-823. doi:10.1113/jphysiol.1997.sp021905

16. Ziemann U, Rothwell JC, Ridding MC. Interaction between intracortical inhibition and facilitation in human motor cortex. *J Physiol*. 1996;496 ( Pt 3)(Pt 3):873-881. doi:10.1113/jphysiol.1996.sp021734

17. Di Lazzaro V, Oliviero A, Profice P, et al. Comparison of descending volleys evoked by transcranial magnetic and electric stimulation in conscious humans. *Electroencephalogr Clin Neurophysiol*. 1998;109(5):397-401. doi:10.1016/s0924-980x(98)00038-1

18. Davila-Pérez P, Pascual-Leone A, Cudeiro J. Effects of transcranial Static Magnetic Stimulation on motor cortex evaluated by different TMS waveforms and current directions. *Neuroscience*. 2019;413:22-30. doi:10.1016/j.neuroscience.2019.05.065

**Supplementary Figures**


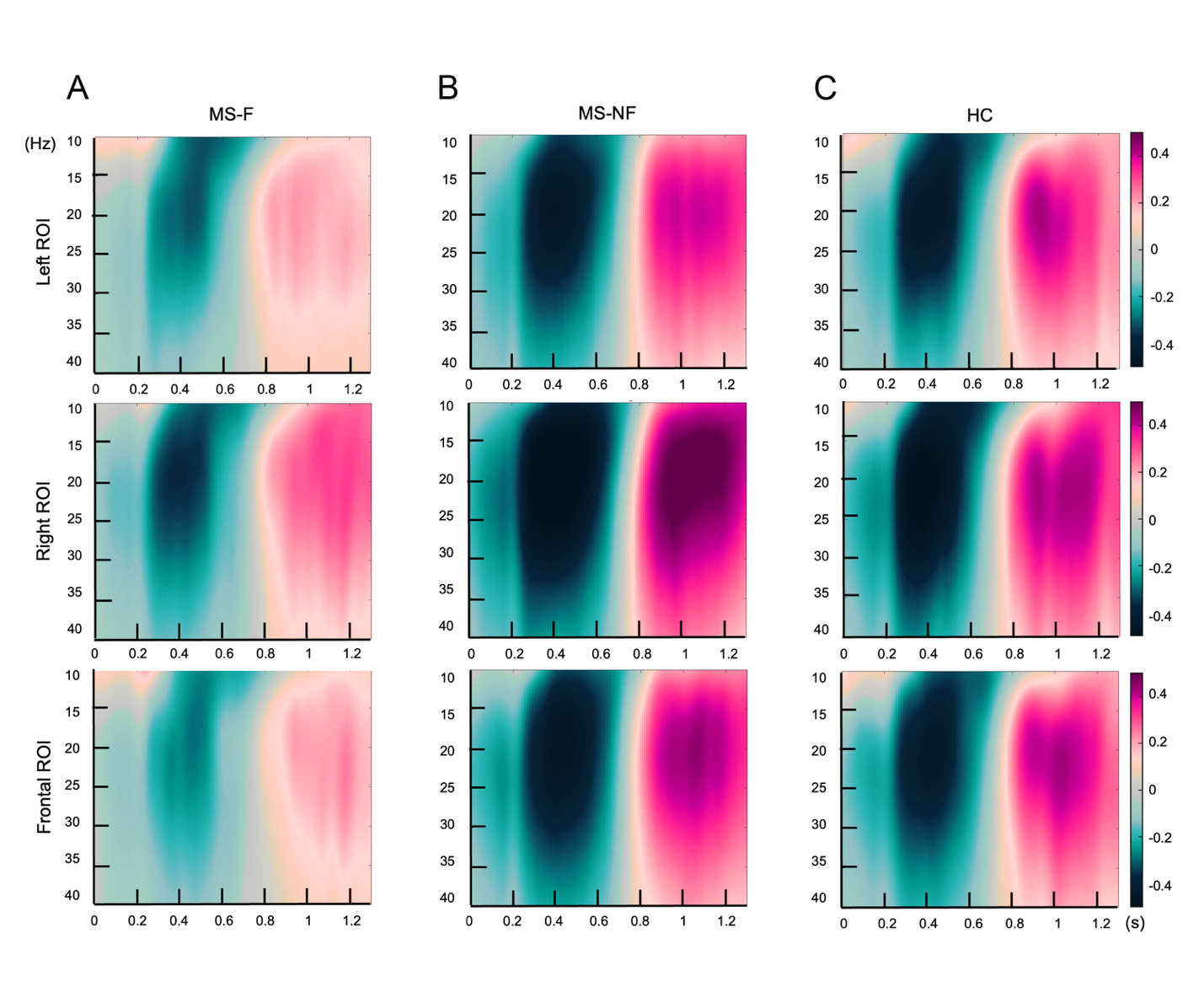
**Supplementary Figure 1.** Time–frequency plots illustrating movement-related beta Event-Related Desynchronization (ERD) (green area) and synchronization (ERS) (magenta area) dynamics (13.5–25 Hz) across the Left, Right, and Frontal Regions of Interest (ROIs) for the Multiple Sclerosis fatigued (MS-F; n = 17) (Panel A), non-fatigued (MS-NF; n = 19) (Panel B), and Healthy Controls (HC; n = 17) (Panel C) groups. Each time–frequency map represents group-averaged beta-band activity computed across individual participants included in the analysis. The x-axis represents time in seconds, with 0 indicating the appearance of the green circle cue. The y-axis denotes frequency in Hz. Color bar indicates amplitude change relative to baseline (unitless).

**
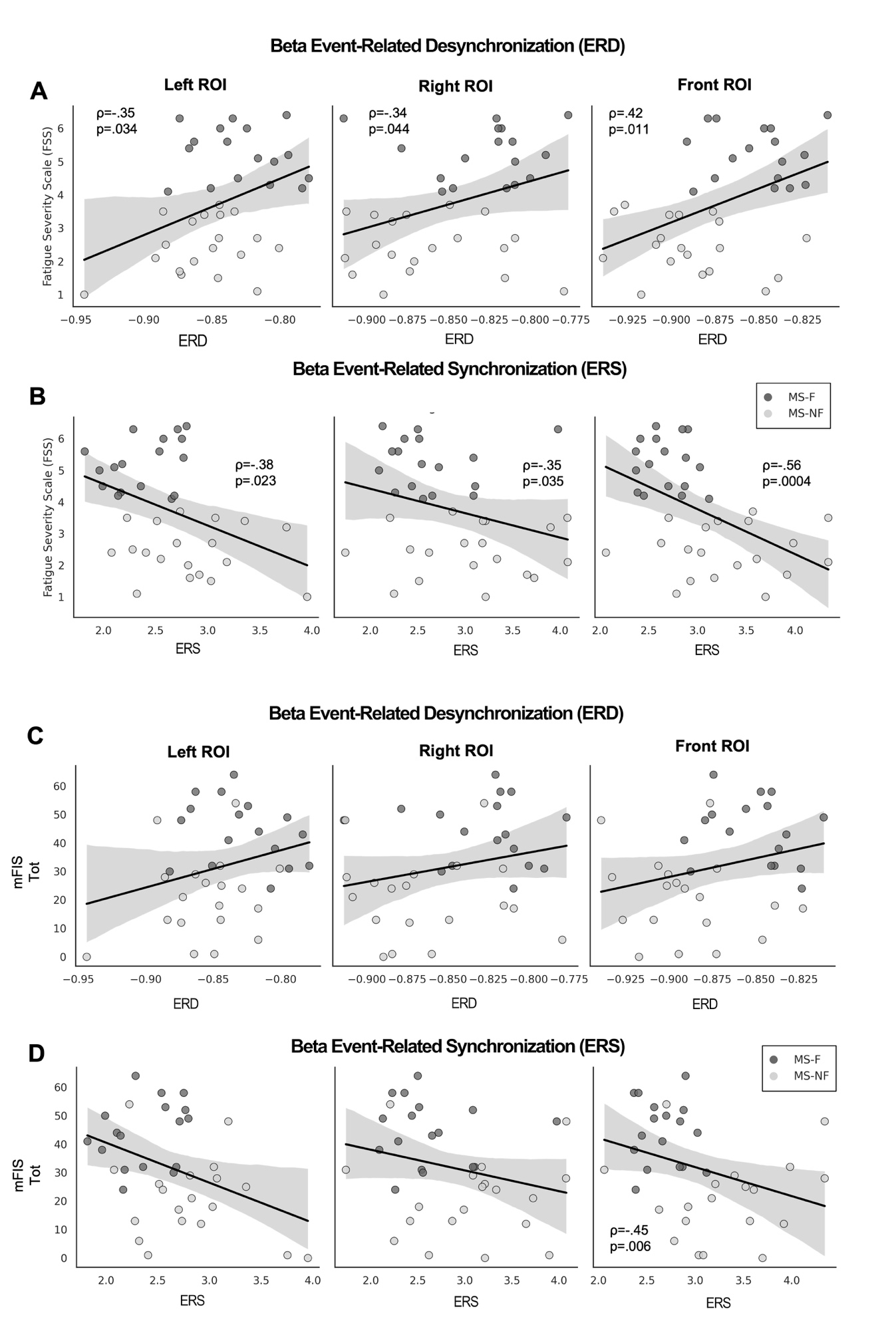
**

**Supplementary Figure 2.** **Associations between beta oscillatory dynamics and fatigue severity.** Scatterplots showing Spearman correlations in MS participants (n= 36) between Fatigue Severity Scale (FSS) scores (Panels A and B) or modified Fatigue Impact Scale total scores (mFIS Total) (Panels C and D) and movement-related beta desynchronization (ERD; A, C) and synchronization (ERS; B, D) within the Left, Right, and Frontal Regions of Interest (ROIs) (unitless). Each data point represents an individual with multiple sclerosis with fatigue (MS-F; dark dots) or without fatigue (MS-NF; light dots). Solid lines indicate regression lines with 95% confidence intervals (Grey areas); Spearman correlation coefficients (*ρ*) and corresponding p-values are reported only for associations with *α*<0.05 (uncorrected). Because three ROIs were tested in parallel for each association, a Bonferroni-corrected significance threshold of *α* = 0.017 (0.05/3) is applied to the family of tests across ROIs.

**Supplementary Tables**

**Supplementary Table 1 | Prescribed medications in the study cohort**

| **Active principle** | **Total** | **MS-F** | **MS-NF** |
| --- | --- | --- | --- |
| Dimethyl fumarate | 18 | 7 | 11 |
| Natalizumab | 4 | 3 | 1 |
| Pregabalin | 2 | 1 | 1 |
| Interferon | 1 | 0 | 1 |
| Cladribine | 3 | 0 | 3 |
| Ocrelizumab | 2 | 2 | 0 |
| Gabapentin | 2 | 2 | 0 |
| Fingolimod | 1 | 0 | 1 |
| Fluticasone furoate | 1 | 0 | 1 |
| Ofatumumab | 1 | 1 | 0 |
| Omalizumab | 1 | 1 | 0 |

**Supplementary Table 1. Prescribed medications in the study cohort.** Table reports the active principles and the number of participants with Multiple Sclerosis (MS) taking each drug in the fatigued (MS-F) and non-fatigued (MS-NF) groups.

**Supplementary Table 2 | Post-hoc comparisons of demographic and clinical measures across groups**

| A. | Comparison | *z* | *p* | BF_10_ |
| --- | --- | --- | --- | --- |
| Age | HC-MS-F | 0.045 | 1.000 | 0.319 |
|  | HC-MS-NF | −0.122 | 1.000 | 0.317 |
|  | MS-F-MS-NF | −0.171 | 1.000 | 0.310 |
| Education | HC-MS-F | 2.912 | **0.011** | 6.935 |
|  | HC-MS-NF | 2.325 | 0.060 | 2.485 |
|  | MS-F-MS-NF | -0.699 | 1.000 | 0.408 |
| BMI | HC-MS-F | -1.584 | 0.339 | 1.371 |
|  | HC-MS-NF | -1.805 | 0.213 | 2.290 |
|  | MS-F-MS-NF | -0.167 | 1.000 | 0.308 |
| BMR | HC-MS-F | 0.390 | 1.000 | 0.344 |
|  | HC-MS-NF | -0.895 | 1.000 | 0.424 |
|  | MS-F-MS-NF | -1.318 | 0.563 | 0.629 |
| R 9-HPT | HC-MS-F | -2.936 | **0.010** | 8.115 |
|  | HC-MS-NF | -0.946 | 1.000 | 0.441 |
|  | MS-F-MS-NF | 2.133 | 0.099 | 1.470 |
| L 9-HPT | HC-MS-F | -2.423 | **0.046** | 0.802 |
|  | HC-MS-NF | -1.086 | 0.832 | 0.466 |
|  | MS-F-MS-NF | 1.455 | 0.437 | 0.311 |
| MADRS | HC-MS-F | −4.017 | **0.0002** | 1402.060 |
|  | HC-MS-NF | −0.966 | 1.000 | 0.727 |
|  | MS-F-MS-NF | 3.247 | **0.003** | 3.226 |

| B. | Comparison | Prior odds | Posterior odds | BF_10_ |
| --- | --- | --- | --- | --- |
| Age | HC-MS-F | 0.587 | 0.187 | 0.319 |
|  | HC-MS-MS-NF | 0.587 | 0.186 | 0.317 |
|  | MS-F-MS-NF | 0.587 | 0.182 | 0.310 |
| Education | HC-MS-F | 0.587 | 4.074 | 6.935 |
|  | HC-MS-NF | 0.587 | 1.460 | 2.485 |
|  | MS-F-MS-NF | 0.587 | 0.240 | 0.408 |
| BMI | HC-MS-F | 0.587 | 0.805 | 1.371 |
|  | HC-MS-NF | 0.587 | 1.345 | 2.290 |
|  | MS-F-MS-NF | 0.587 | 0.181 | 0.308 |
| BMR | HC-MS-F | 0.587 | 0.202 | 0.344 |
|  | HC-MS-NF | 0.587 | 0.249 | 0.424 |
|  | MS-F-MS-NF | 0.587 | 0.369 | 0.629 |
| R 9-HPT | HC-MS-F | 0.587 | 4.767 | 8.115 |
|  | HC-MS-NF | 0.587 | 0.259 | 0.441 |
|  | MS-F-MS-NF | 0.587 | 0.863 | 1.470 |
| L 9-HPT | HC-MS-F | 0.587 | 0.471 | 0.802 |
|  | HC-MS-NF | 0.587 | 0.274 | 0.466 |
|  | MS-F-MS-NF | 0.587 | 0.183 | 0.311 |
| MADRS | HC-MS-F | 0.587 | 823.572 | 1402.060 |
|  | HC-MS-NF | 0.587 | 0.427 | 0.727 |
|  | MS-F-MS-NF | 0.587 | 1.895 | 3.226 |

**Supplementary Table 2.** Post-hoc pairwise comparisons of demographic and clinical measures among MS-F, MS-NF, and HC. *z* denotes the standardized test statistic for pairwise comparisons; *p* denotes the p-value; BF₁₀ denotes the Bayes factor indicating the strength of evidence in favor of the alternative hypothesis over the null hypothesis. Significant p-values are shown in bold.

**Abbreviations:** HC, healthy controls; MS-F, multiple sclerosis with fatigue; MS-NF, multiple sclerosis without fatigue; BMI, body mass index; BMR, basal metabolic rate; 9-HPT, Nine-Hole Peg Test; MADRS, Montgomery-Åsberg Depression Rating Scale; BF₁₀, Bayes factor in favor of the alternative hypothesis.

**Supplementary Table 3 | Kruskal-Wallis test results on cognitive performance**

| Test | *H* | *p* | n |
| --- | --- | --- | --- |
| SRT–LTS | 2.094 | 0.351 | 57 |
| SRT–CLTR | 2.112 | 0.348 | 57 |
| SPART | 1.227 | 0.542 | 58 |
| SDMT | 0.730 | 0.694 | 58 |
| PASAT 3 | 0.913 | 0.633 | 57 |
| PASAT 2 | 2.816 | 0.245 | 57 |
| SRT–D | 0.367 | 0.833 | 57 |
| WLG | 4.625 | 0.099 | 58 |

**Supplementary Table 3.** Results of the Kruskal-Wallis tests across cognitive performance indexes measured with the Rao Brief Repeatable Battery. For each test, the Kruskal-Wallis H statistic (H) and corresponding p-value (*p*) are reported, together with sample sizes (n).

**Abbreviations: Selective Reminding Test–Long Term Storage (SRT-LTS)**, **Selective Reminding Test–Consistent Long-Term Retrieval (SRT-CLTR)**, **Spatial Recall Test (SPART)**, **Symbol Digit Modalities Test (SDMT)**, **Paced Auditory Serial Addition Test 3-second (PASAT 3)**, **Paced Auditory Serial Addition Test 2-second (PASAT 2)**, **Selective Reminding Test–Delayed Recall (SRT-D)**, and **Word List Generation (WLG)**.

**Supplementary Table 4 | Group comparisons for blood biomarkers of inflammation**

| **Dependent Variable** | **H** | **p** | **BF_10_** | **Post-hoc** | **z** | **p** | **BF_10_ (post-hoc)** |
| --- | --- | --- | --- | --- | --- | --- | --- |
| MS-NFL | 5.513 | 0.064 | 0.895 | HC vs MS-F | -2.18 | 0.044 | 1.39 |
|  |  |  |  | HC vs MS-NF | -2.14 | 0.049 | 3.70 |
|  |  |  |  | MS-F vs MS-NF | 0.15 | 1.000 | 0.39 |
| GFAP | 0.041 | 0.980 | 0.226 | HC vs MS-F | -0.20 | 1.000 | 0.50 |
|  |  |  |  | HC vs MS-NF | -0.17 | 1.000 | 0.49 |
|  |  |  |  | MS-F vs MS-NF | 0.05 | 1.000 | 0.33 |
| IL-4 | 5.365 | 0.068 | 0.837 | HC vs MS-F | 2.30 | 0.032 | 1.42 |
|  |  |  |  | HC vs MS-NF | 1.27 | 0.306 | 1.19 |
|  |  |  |  | MS-F vs MS-NF | -1.30 | 0.291 | 0.39 |
| IL-2 | 4.359 | 0.113 | 0.607 | HC vs MS-F | 2.08 | 0.056 | 1.13 |
|  |  |  |  | HC vs MS-NF | 1.48 | 0.210 | 0.52 |
|  |  |  |  | MS-F vs MS-NF | -0.79 | 0.641 | 0.76 |
| IP-10 | 2.318 | 0.314 | 0.351 | HC vs MS-F | 1.47 | 0.213 | 0.72 |
|  |  |  |  | HC vs MS-NF | 0.64 | 0.788 | 0.55 |
|  |  |  |  | MS-F vs MS-NF | -1.03 | 0.453 | 0.37 |
| IL-1β | 4.040 | 0.133 | 0.748 | HC vs MS-F | 1.92 | 0.082 | 1.79 |
|  |  |  |  | HC vs MS-NF | 0.77 | 0.662 | 0.52 |
|  |  |  |  | MS-F vs MS-NF | -1.42 | 0.234 | 0.65 |
| TNF-α | 3.643 | 0.162 | 0.553 | HC vs MS-F | 1.83 | 0.101 | 1.14 |
|  |  |  |  | HC vs MS-NF | 0.76 | 0.675 | 0.54 |
|  |  |  |  | MS-F vs MS-NF | -1.33 | 0.277 | 0.51 |
| MCP-1 | 1.311 | 0.519 | 0.802 | HC vs MS-F | 1.01 | 0.466 | 0.75 |
|  |  |  |  | HC vs MS-NF | 1.06 | 0.435 | 1.58 |
|  |  |  |  | MS-F vs MS-NF | 0.00 | 1.000 | 0.38 |
| IL-17A | 2.503 | 0.286 | 0.579 | HC vs MS-F | 1.57 | 0.176 | 1.79 |
|  |  |  |  | HC vs MS-NF | 1.17 | 0.361 | 0.58 |
|  |  |  |  | MS-F vs MS-NF | -0.53 | 0.897 | 0.43 |
| IL-6 | 4.260 | 0.119 | 0.250 | HC vs MS-F | 1.93 | 0.081 | 1.57 |
|  |  |  |  | HC vs MS-NF | 1.80 | 0.107 | 0.41 |
|  |  |  |  | MS-F vs MS-NF | -0.23 | 1.000 | 0.36 |
| IL-10 | 8.450 | **0.015** | 0.724 | HC vs MS-F | 2.91 | **0.005** | 16.42 |
|  |  |  |  | HC vs MS-NF | 1.83 | 0.101 | 0.51 |
|  |  |  |  | MS-F vs MS-NF | -1.38 | 0.250 | 0.51 |
| IFN-γ | 3.095 | 0.213 | 1.771 | HC vs MS-F | 1.70 | 0.133 | 2.79 |
|  |  |  |  | HC vs MS-NF | 0.75 | 0.680 | 0.93 |
|  |  |  |  | MS-F vs MS-NF | -1.18 | 0.357 | 0.67 |
| IL-12p70 | 10.014 | **0.007** | 1.143 | HC vs MS-F | 3.06 | **0.003** | 6.75 |
|  |  |  |  | HC vs MS-NF | 2.59 | **0.015** | 0.88 |
|  |  |  |  | MS-F vs MS-NF | -0.69 | 0.739 | 0.40 |
| IL-8 | 5.401 | 0.067 | 1.849 | HC vs MS-F | 2.18 | 0.044 | 7.26 |
|  |  |  |  | HC vs MS-NF | 0.77 | 0.658 | 0.58 |
|  |  |  |  | MS-F vs MS-NF | -1.72 | 0.127 | 1.14 |
| TGF-β1 (Free Active) | 1.413 | 0.493 | 0.508 | HC vs MS-F | 0.74 | 0.691 | 0.88 |
|  |  |  |  | HC vs MS-NF | -0.24 | 1.000 | 0.59 |
|  |  |  |  | MS-F vs MS-NF | -1.17 | 0.365 | 0.45 |

**Supplementary Table 4.** Main group effects (Kruskal-Wallis *H*, *p*, and Bayesian BF₁₀) and post-hoc pairwise comparisons (Dunn’s test: *z*, *p*; Bayesian BF₁₀) for comparisons among healthy controls (HC), multiple sclerosis with fatigue (MS-F), and multiple sclerosis without fatigue (MS-NF) across blood-derived inflammation- and neurodegeneration-related biomarkers. BF₁₀ denotes the Bayes factor indicating the strength of evidence in favor of the alternative hypothesis over the null hypothesis. Significant *p*-values are shown in bold.

**Abbreviations:** Neurofilament light chain (NfL); glial fibrillary acidic protein (GFAP); interleukin-4 (IL-4); interleukin-2 (IL-2); interferon gamma–induced protein 10 (IP-10); interleukin-1 beta (IL-1β); tumor necrosis factor alpha (TNF-α); monocyte chemoattractant protein-1 (MCP-1); interleukin-17A (IL-17A); interleukin-6 (IL-6); interleukin-10 (IL-10); interferon gamma (IFN-γ); interleukin-12 p70 heterodimer (IL-12p70); interleukin-8 (IL-8); HC, healthy controls; MS-F, multiple sclerosis with fatigue; MS-NF, multiple sclerosis without fatigue; BF₁₀, Bayes factor in favor of the alternative hypothesis.

# **Supplementary Table 5 | Bayesian ANOVA and post-hoc comparisons for structural MRI measures**

|  | BF_10_ | Comparison | Prior Odds | Posterior Odds | Post-hoc  BF_10_ |
| --- | --- | --- | --- | --- | --- |
| N. Lesions | 58.76 | HC vs MS-F | 0.59 | 111.08 | 189.10 |
|  |  | HC vs MS-NF | 0.59 | 50.50 | 85.97 |
|  |  | MS-F vs MS_NF | 0.59 | 0.18 | 0.31 |
| Vol. Lesions | 3.77 | HC vs MS-F | 0.59 | 743.96 | 1266.53 |
|  |  | HC vs MS_NF | 0.59 | 1.57 | 2.67 |
|  |  | MS-F vs MS_NF | 0.59 | 0.18 | 0.31 |
| GM | 0.42 | HC vs MS-F | 0.59 | 0.46 | 0.78 |
|  |  | HC vs MS-NF | 0.59 | 0.58 | 0.99 |
|  |  | MS-F vs MS-NF | 0.59 | 0.18 | 0.31 |
| WM | 2.10 | HC vs MS-F | 0.59 | 2.47 | 4.20 |
|  |  | HC vs MS-NF | 0.59 | 0.26 | 0.45 |
|  |  | MS-F vs MS-NF | 0.59 | 0.78 | 1.335 |
| Thalamus | 68.56 | HC vs MS-F | 0.59 | 75.65 | 128.79 |
|  |  | HC vs MS-NF | 0.59 | 25.54 | 43.470 |
|  |  | MS-F vs MS-NF | 0.59 | 0.22 | 0.38 |
| Caudate | 1.79 | HC vs MS-F | 0.59 | 2.66 | 4.53 |
|  |  | HC vs MS-NF | 0.59 | 0.33 | 0.55 |
|  |  | MS-F vs MS-NF | 0.59 | 0.49 | 0.84 |

**Supplementary Table 5.** Bayesian ANOVA and post-hoc comparisons for the number and volume of lesions and for total grey matter (GM), white matter (WM), thalamus, and caudate volumes. The table reports Bayes factors in favor of the alternative hypothesis from the omnibus Bayesian ANOVA (BF₁₀), as well as prior odds, posterior odds, and Bayes factors from post-hoc pairwise comparisons among healthy controls (HC), multiple sclerosis with fatigue (MS-F), and multiple sclerosis without fatigue (MS-NF). Posterior odds were derived assuming a prior probability of 0.5 for the null hypothesis across comparisons.

**Supplementary Table 6 | Mediation analyses results for the WM, thalamic and caudate volumes**

**Mediator: MADRS**

| **Predicted Variable** | **Effect Type** | **Estimate (SE)** | **z** | **p** | **95% CI Lower** | **95% CI Upper** |
| --- | --- | --- | --- | --- | --- | --- |
| **White Matter volume** |  |  |  |  |  |  |
|  | Direct Effect | -16.03(10.94) | -1.46 | 0.143 | -37.56 | 2.06 |
| Dummy (MS-F=1, HC=0) | Indirect Effect | -6.326(7.358) | -0.86 | 0.390 | -23.97 | 5.86 |
|  | Total Effect | -22.35(8.26) | -2.71 | **0.007** | -38.73 | -5.72 |
| **Thalamus volume** |  |  |  |  |  |  |
| Dummy (MS-F=1, HC=0) | Direct Effect | -1.85 (0.63) | -2.96 | **0.003** | -3.11 | -0.80 |
|  | Indirect Effect | -0.16(0.42) | -0.38 | 0.71 | -0.91 | 0.61 |
|  | Total Effect | -2.01(0.47) | -4.30 | **0.00002** | -2.96 | -1.06 |
| **Caudate volume** |  |  |  |  |  |  |
| Dummy (MS-F=1, HC=0) | Direct Effect | -0.90(0.40) | -2.23 | **0.026** | -1.57 | -0.12 |
|  | Indirect Effect | 0.07(0.27) | 0.25 | 0.80 | -0.38 | 0.65 |
|  | Total Effect | -0.83(0.30) | -2.75 | **0.006** | -1.40 | -0.21 |

**Mediator: Right 9-HPT**

| **Predicted Variable** | **Effect Type** | **Estimate (SE)** | **z** | **p** | **95% CI Lower** | **95% CI Upper** |
| --- | --- | --- | --- | --- | --- | --- |
| **White Matter volume** |  |  |  |  |  |  |
|  | Direct Effect | -15.54(8.99) | -1.729 | 0.084 | -33.37 | 4.45 |
|  | Indirect Effect | -6.815(4.69) | -1.453 | 0.146 | -21.34 | 2.52 |
|  | Total Effect | -22.352(8.26) | -2.705 | **0.007** | -40.18 | -6.57 |
| **Thalamus volume** |  |  |  |  |  |  |
| Dummy (MS-F=1, HC=0) | Direct Effect | -1.668(0.51) | -3.28 | **0.001** | -2.72 | -0.72 |
|  | Indirect Effect | -0.34(0.26) | -1.337 | 0.181 | -1.52 | 0.32 |
|  | Total Effect | -2.008(0.47) | -4.303 | **0.00017** | -3.02 | -1.19 |
| **Caudate volume** |  |  |  |  |  |  |
| Dummy (MS-F=1, HC=0) | Direct Effect | -0.646(0.33) | -1.934 | 0.053 | -1.32 | 0.03 |
|  | Indirect Effect | -0.187(0.16) | -1.139 | 0.255 | -0.57 | 0.04 |
|  | Total Effect | -0.832(0.30) | -2.747 | **0.006** | -1.45 | -0.26 |

**Mediator: Left 9-HPT**

| **Predicted Variable** | **Effect Type** | **Estimate (SE)** | **z** | **p** | **95% CI Lower** | **95% CI Upper** |
| --- | --- | --- | --- | --- | --- | --- |
| **White Matter volume** |  |  |  |  |  |  |
| Dummy (MS-F=1, HC=0) | Direct Effect | -23.256(8.52) | -2.728 | **0.006** | -42.60 | -4.76 |
|  | Indirect Effect | 0.904(2.24) | 0.403 | 0.687 | -5.01 | 10.04 |
|  | Total Effect | -22.35(8.26) | -2.705 | **0.007** | -39.49 | -6.91 |
| **Thalamus volume** |  |  |  |  |  |  |
| Dummy (MS-F=1, HC=0) | Direct Effect | -2.103(0.48) | -4.372 | **0.000012** | -3.12 | -1.26 |
|  | Indirect Effect | 0.095(0.14) | 0.665 | 0.506 | -0.13 | 0.59 |
|  | Total Effect | -2.008(0.47) | -4.303 | **0.000017** | -.2.97 | -1.16 |
| **Caudate volume** |  |  |  |  |  |  |
| Dummy (MS-F=1, HC=0) | Direct Effect | -0.69(0.30) | -2.30 | **0.021** | -1.27 | -0.01 |
|  | Indirect Effect | -0.140(0.12) | -1.195 | 0.232 | -0.78 | 0.01 |
|  | Total Effect | 1.943(1.22) | 1.59 | 0.111 | -0.76 | 4.06 |

**Supplementary Table 6.** Mediation analysis results for the white matter (WM), thalamus, and caudate volumes. The table reports the direct, indirect, and total effects of group on regional brain volumes, mediated by the Montgomery–Åsberg Depression Rating Scale (MADRS) or the Nine-Hole Peg Test (9-HPT) (Left and Right hand) scores. Values are presented as estimates with standard errors (SE), *z*-values, *p*-values, and 95% confidence intervals (CI). Significant *p*-values are shown in bold. Abbreviations: HC, healthy controls; MS-F, multiple sclerosis with fatigue.

**Supplementary Table 7 | Mediation analyses results for the integrity of white matter tracts**

**Mediator: MADRS**

| **Predicted Variable** | **Effect Type** | **Estimate (SE)** | **z** | **p** | **95% CI Lower** | **95% CI Upper** |
| --- | --- | --- | --- | --- | --- | --- |
| **Left CST** |  |  |  |  |  |  |
| Dummy (MS-F=1, HC=0) | Direct Effect | 0.007 (0.025) | 0.280 | 0.780 | -0.058 | 0.057 |
|  | Indirect Effect | -0.003 (0.016) | -0.207 | 0.836 | -0.037 | 0.035 |
|  | Total Effect | 0.004 (0.019) | 0.191 | 0.849 | -0.035 | 0.038 |
| **Right CST** |  |  |  |  |  |  |
| Dummy (MS-F=1, HC=0) | Direct Effect | -0.079 (0.029) | -2.679 | **0.007** | -0.138 | -0.030 |
|  | Indirect Effect | -0.005 (0.020) | -0.272 | 0.785 | -0.037 | 0.027 |
|  | Total Effect | -0.084 (0.022) | -3.818 | **0.0001** | -0.132 | -0.038 |
| **Right Thalamic Radiation** |  |  |  |  |  |  |
| Dummy (MS-NF=1, HC=0) | Direct Effect | -0.066 (0.026) | -2.545 | **0.011** | -0.114 | -0.014 |
|  | Indirect Effect | -0.001 (0.010) | -0.090 | 0.928 | -0.031 | 0.028 |
|  | Total Effect | -0.067 (0.024) | -2.781 | **0.005** | -0.113 | -0.018 |
| **Left SLF** |  |  |  |  |  |  |
| Dummy (MS-NF=1, HC=0) | Direct Effect | -0.032 (0.026) | -1.225 | 0.221 | -0.094 | 0.025 |
|  | Indirect Effect | 0.005 (0.010) | 0.485 | 0.628 | -0.026 | 0.017 |
|  | Total Effect | -0.027 (0.024) | -1.118 | 0.264 | -0.078 | 0.019 |
| **Right SLF** |  |  |  |  |  |  |
| Dummy (MS-NF=1, HC=0) | Direct Effect | -0.072 (0.020) | -3.526 | **0.0004** | -0.114 | -0.028 |
|  | Indirect Effect | -0.012 (0.009) | -1.351 | 0.177 | -0.045 | 0.001 |
|  | Total Effect | -0.084 (0.020) | -4.315 | **0.00002** | -0.125 | -0.044 |

**Mediator: Right 9-HPT**

| **Predicted Variable** | **Effect Type** | **Estimate (SE)** | **z** | **p** | **95% CI Lower** | **95% CI Upper** |
| --- | --- | --- | --- | --- | --- | --- |
| **Right CST** |  |  |  |  |  |  |
| Dummy (MS-F=1, HC=0) | Direct Effect | -0.084 (0.025) | -3.382 | **0.0007** | -0.130 | -0.036 |
|  | Indirect Effect | 0.000 (0.008) | 0.002 | 0.998 | -0.023 | 0.019 |
|  | Total Effect | -0.084 (0.022) | -3.818 | **0.0001** | -0.132 | -0.041 |
| **Left CST** |  |  |  |  |  |  |
| Dummy (MS-F=1, HC=0) | Direct Effect | 0.004 (0.021) | 0.211 | 0.833 | -0.035 | 0.043 |
|  | Indirect Effect | 0.000 (0.009) | -0.090 | 0.928 | -0.022 | 0.020 |
|  | Total Effect | 0.004 (0.019) | 0.191 | 0.849 | -0.033 | 0.039 |
| **Right Thalamic Radiation** |  |  |  |  |  |  |
| Dummy (MS-NF=1, HC=0) | Direct Effect | -0.067 (0.025) | -2.644 | **0.008** | -0.115 | -0.008 |
|  | Indirect Effect | 0.00001 (0.008) | 0.002 | 0.998 | -0.023 | 0.019 |
|  | Total Effect | -0.067 (0.024) | -2.781 | **0.005** | -0.109 | -0.013 |
| **Left SLF** |  |  |  |  |  |  |
| Dummy (MS-NF=1, HC=0) | Direct Effect | -0.021 (0.025) | -0.838 | 0.402 | -0.071 | 0.038 |
|  | Indirect Effect | -0.006 (0.008) | -0.707 | 0.480 | -0.042 | 0.006 |
|  | Total Effect | -0.027 (0.024) | -1.118 | 0.264 | -0.072 | 0.021 |
| **Right SLF** |  |  |  |  |  |  |
| Dummy (MS-NF=1, HC=0) | Direct Effect | -0.069 (0.019) | -3.610 | **0.000306** | -0.105 | -0.031 |
|  | Indirect Effect | -0.016 (0.010) | -1.631 | 0.103 | -0.048 | -0.001 |
|  | Total Effect | -0.084 (0.020) | -4.315 | **0.000016** | -0.127 | -0.047 |

**Left Mediator: 9-HPT**

| **Predicted Variable** | **Effect Type** | **Estimate (SE)** | **z** | **p** | **95% CI Lower** | **95% CI Upper** |
| --- | --- | --- | --- | --- | --- | --- |
| **Right CST** |  |  |  |  |  |  |
| Dummy (MS-F=1, HC=0) | Direct Effect | -0.089 (0.023) | -3.914 | **0.00009** | -0.138 | -0.045 |
|  | Indirect Effect | 0.004 (0.006) | 0.685 | 0.493 | -0.011 | 0.026 |
|  | Total Effect | -0.084 (0.022) | -3.818 | **0.0001** | -0.130 | -0.042 |
| **Left CST** |  |  |  |  |  |  |
| Dummy (MS-F=1, HC=0) | Direct Effect | 0.0005 (0.019) | -0.026 | 0.980 | -0.041 | 0.035 |
|  | Indirect Effect | 0.004 (0.006) | 0.731 | 0.465 | -0.002 | 0.033 |
|  | Total Effect | 0.004 (0.019) | 0.191 | 0.849 | -0.032 | 0.040 |
| **Right Thalamic Radiation** |  |  |  |  |  |  |
| Dummy (MS-NF=1, HC=0) | Direct Effect | -0.067 (0.024) | -2.783 | **0.005** | -0.116 | -0.010 |
|  | Indirect Effect | 0.0002 (0.002) | -0.081 | 0.935 | -0.012 | 0.010 |
|  | Total Effect | -0.067 (0.024) | -2.781 | **0.005** | -0.116 | -0.012 |
| **Left SLF** |  |  |  |  |  |  |
| Dummy (MS-NF=1, HC=0) | Direct Effect | -0.027 (0.024) | -1.121 | 0.262 | -0.077 | 0.024 |
|  | Indirect Effect | 0.0002 (0.003) | -0.051 | 0.959 | -0.010 | 0.010 |
|  | Total Effect | -0.027 (0.024) | -1.118 | 0.264 | -0.074 | 0.023 |
| **Right SLF** |  |  |  |  |  |  |
| Dummy (MS-NF=1, HC=0) | Direct Effect | -0.085 (0.019) | -4.392 | **0.00001** | -0.127 | -0.051 |
|  | Indirect Effect | 0.0003 (0.003) | 0.084 | 0.933 | -0.012 | 0.009 |
|  | Total Effect | -0.084 (0.020) | -4.315 | **0.00002** | -0.122 | -0.050 |

**Supplementary Table 7.** Mediation analysis results for white matter fractional anisotropy (FA) values. The table reports direct, indirect, and total effects of group on FA values, with mediation by depressive symptoms assessed using the Montgomery-Åsberg Depression Rating Scale (MADRS) or motor performance assessed using the Nine-Hole Peg Test (9-HPT; left and right hand). Values are presented as estimates with standard errors (SE), *z*-values, *p*-values, and 95% confidence intervals (CI). Significant *p*-values are shown in bold.

**Abbreviations:** Left CST, left corticospinal tract; Right CST, right corticospinal tract; L SLF, left superior longitudinal fasciculus; R SLF, right superior longitudinal fasciculus; HC, healthy controls; MS-F, multiple sclerosis with fatigue.

**Supplementary Table 8 |** Bayesian analyses on beta ERD, ERS and modulation depth

| BF_10_ | **ERD** | | | **ERS** | | | **Modulation depth** | | |
| --- | --- | --- | --- | --- | --- | --- | --- | --- | --- |
|  | Left | Right | Frontal | Left | Right | Frontal | Left | Right | Frontal |
| Null Model | 0.5 | 0.5 | 0.5 | 0.5 | 0.5 | 0.5 | 0.5 | 0.5 | 0.5 |
| Main Group | 1.713 | **5.528** | **186.67** | 1.979 | 1.295 | **101.94** | 2.18 | 1.46 | **129.41** |
| HC vs. MSF | 1.503 | **3.481** | **26.66** | 1.816 | 0.598 | **166.40** | 1.93 | 0.66 | **189.04** |
| HC vs. NF | 0.348 | 0.389 | 0.67 | 0.326 | 0.596 | 0.379 | 0.326 | 0.59 | 0.388 |
| MSF vs. NF | **3.246** | **6.931** | **114.60** | **11.43** | 2.38 | **115.50** | **12.542** | 2.67 | **115.50** |

**Supplementary Table 8.** Bayesian ANOVA and post-hoc results for group effects on beta event-related desynchronization (ERD), event-related synchronization (ERS), and modulation depth across left, right, and frontal regions of interest (ROIs). The first two rows report Bayes factors in favor of the alternative hypothesis (BF₁₀) for the null model and the main effect of group. The remaining rows report BF₁₀ values from post-hoc pairwise comparisons among healthy controls (HC), multiple sclerosis with fatigue (MS-F), and multiple sclerosis without fatigue (MS-NF). Bayes factors indicating moderate to decisive evidence for the alternative hypothesis (BF₁₀ ≥ 3) are shown in bold.

**Abbreviations:** ERD, event-related desynchronization; ERS, event-related synchronization; ROI, region of interest; HC, healthy controls; MS-F, multiple sclerosis with fatigue; MS-NF, multiple sclerosis without fatigue; BF₁₀, Bayes factor in favor of the alternative hypothesis.

# **Supplementary Table 9 | Mediation analysis results for beta ERD and ERS**

|  | **MADRS** | **Estimate (SE)** | ***z*** | ***p*** | **95% CI Lower** | **95% CI Upper** |
| --- | --- | --- | --- | --- | --- | --- |
| ERD  Left ROI | Direct Effect | 0.032  (0.015) | 2.161 | **0.031** | 0.003 | 0.061 |
|  | Indirect Effect | -0.009 (0.010) | -0.928 | 0.354 | -0.028 | 0.01 |
|  | Total Effect | 0.023 (0.011) | 2.127 | **0.033** | 0.002 | 0.044 |
| ERD  Right ROI | Direct Effect | 0.028 (0.011) | 2.7 | **0.007** | 0.008 | 0.049 |
|  | Indirect Effect | -0.013 (0.009) | -1.362 | 0.173 | -0.031 | 0.006 |
|  | Total Effect | 0.028 (0.011) | 2.7 | 0.007 | 0.008 | 0.049 |
| ERD  Frontal ROI | Direct Effect | 0.027 (0.007) | 3.7 | **0.0002** | 0.013 | 0.041 |
|  | Indirect Effect | -0.005 (0.007) | -0.736 | 0.462 | -0.018 | 0.008 |
|  | Total Effect | 0.027 (0.007) | 3.7 | **0.0002** | 0.013 | 0.041 |
| ERS  Left ROI | Direct Effect | -0.411 (0.188) | -2.194 | **0.028** | -0.779 | -0.044 |
|  | Indirect Effect | -0.020 (0.171) | -0.118 | 0.906 | -0.355 | 0.315 |
|  | Total Effect | -0.411 (0.188) | -2.194 | **0.028** | -0.779 | -0.044 |
| ERS  Right ROI | Direct Effect | -0.214 (0.157) | -1.36 | 0.174 | -0.521 | 0.094 |
|  | Indirect Effect | 0.190 (0.141) | 1.351 | 0.177 | -0.086 | 0.466 |
|  | Total Effect | -0.214 (0.157) | -1.36 | 0.174 | -0.521 | 0.094 |
| ERS  Frontal ROI | Direct Effect | -0.570 (0.162) | -3.522 | **0.0004** | -0.888 | -0.253 |
|  | Indirect Effect | 0.046 (0.106) | 0.434 | 0.664 | -0.162 | 0.255 |
|  | Total Effect | -0.524 (0.117) | -4.492 | **0.000006** | -0.753 | -0.296 |

|  | **9-HPT** | **Estimate (SE)** | ***z*** | ***p*** | **95% CI Lower** | **95% CI Upper** |
| --- | --- | --- | --- | --- | --- | --- |
| ERD  Left ROI | Direct Effect | 0.026 (0.012) | 2.200 | **0.028** | 0.003 | 0.050 |
|  | Indirect Effect | -0.004 (0.006) | -0.735 | 0.463 | -0.015 | 0.007 |
|  | Total Effect | 0.022 (0.011) | 2.100 | **0.036** | 0.001 | 0.043 |
| ERD  Right ROI | Direct Effect | 0.035 (0.012) | 2.969 | **0.003** | 0.012 | 0.058 |
|  | Indirect Effect | -0.007 (0.006) | -1.187 | 0.235 | -0.017 | 0.004 |
|  | Total Effect | 0.028 (0.010) | 2.691 | **0.007** | 0.008 | 0.049 |
| ERD  Frontal ROI | Direct Effect | 0.031 (0.008) | 3.849 | **0.0001** | 0.015 | 0.047 |
|  | Indirect Effect | -0.004 (0.004) | -1.099 | 0.272 | -0.012 | 0.003 |
|  | Total Effect | 0.027 (0.007) | 3.706 | **0.0002** | 0.013 | 0.041 |
| ERS  Left ROI | Direct Effect | -0.410 (0.213) | -1.928 | 0.054 | -0.826 | 0.007 |
|  | Indirect Effect | -0.003 (0.094) | -0.030 | 0.976 | -0.188 | 0.182 |
|  | Total Effect | -0.413 (0.187) | -2.203 | **0.028** | -0.780 | -0.045 |
| ERS  Right ROI | Direct Effect | -0.263 (0.178) | -1.478 | 0.140 | -0.612 | 0.086 |
|  | Indirect Effect | 0.056 (0.080) | 0.704 | 0.482 | -0.100 | 0.212 |
|  | Total Effect | -0.207 (0.157) | -1.317 | 0.188 | -0.515 | 0.101 |
| ERS  Frontal ROI | Direct Effect | -0.587 (0.130) | -4.527 | **0.000006** | -0.841 | -0.333 |
|  | Indirect Effect | 0.062 (0.061) | 1.021 | 0.307 | -0.057 | 0.181 |
|  | Total Effect | -0.525 (0.116) | -4.514 | **0.000006** | -0.753 | -0.297 |

**Supplementary Table 9.** Mediation analysis results for beta Event-Related Desynchronization (ERD) and Event-Related Synchronization (ERS) across Left, Right, and Frontal regions of interest (ROIs), examining the roles of depressive symptoms assessed with the Montgomery-Åsberg Depression Rating Scale (MADRS) and manual dexterity assessed with the right-hand Nine-Hole Peg Test (9-HPT) as mediators. Group was coded as a dummy variable contrasting multiple sclerosis with fatigue (MS-F) and healthy controls (HC). The table reports direct, indirect, and total effects of group differences on ERD and ERS, with mediation by MADRS (panel A) and right-hand 9-HPT (panel B). Values are presented as estimates with standard errors, corresponding *z*-values, *p*-values, and 95% confidence intervals (CI). Significant *p*-values are shown in bold.

# **Supplementary Table 10** | Spearman correlation analyses between clinical measures and beta ERD and ERS magnitude

| **Measure Pair** | | ***ρ*** | ***p*** | ***95% CI*** | |
| --- | --- | --- | --- | --- | --- |
| FSS | ERD Left ROI | 0.354 | **0.034** | 0.031 | 0.615 |
|  | ERD Right ROI | 0.338 | **0.044** | -0.059 | 0.668 |
|  | ERD Frontal ROI | 0.419 | **0.011** | 0.116 | 0.650 |
|  | ERS Left ROI | -0.377 | **0.023** | -0.628 | -0.054 |
|  | ERS Right ROI | -0.353 | **0.035** | -0.655 | -0.013 |
|  | ERS Frontal ROI | -0.556 | **0.0004** | -0.722 | -0.302 |
| mFIS Tot | ERD Left ROI | 0.252 | 0.139 | -0.060 | 0.521 |
|  | ERD Right ROI | 0.227 | 0.183 | -0.118 | 0.521 |
|  | ERD Frontal ROI | 0.274 | 0.106 | -0.027 | 0.544 |
|  | ERS Left ROI | -0.303 | 0.072 | -0.573 | 0.008 |
|  | ERS Right ROI | -0.332 | 0.048 | -0.593 | -0.027 |
|  | ERS Frontal ROI | -0.445 | **0.006** | -0.664 | -0.160 |
| mFIS Cog | ERD Left ROI | 0.247 | 0.147 | -0.058 | 0.528 |
|  | ERD Right ROI | 0.180 | 0.294 | -0.141 | 0.480 |
|  | ERD Frontal ROI | 0.218 | 0.201 | -0.100 | 0.501 |
|  | ERS Left ROI | -0.224 | 0.189 | -0540 | 0.088 |
|  | ERS Right ROI | -0.300 | 0.076 | -0.574 | 0.005 |
|  | ERS Frontal ROI | -0.395 | **0.017** | -0.614 | -0.101 |
| mFIS Phy | ERD Left ROI | 0.160 | 0.350 | -0.148 | 0.448 |
|  | ERD Right ROI | 0.161 | 0.348 | -0.168 | 0.484 |
|  | ERD Frontal ROI | 0.252 | 0.139 | -0.034 | 0.525 |
|  | ERS Left ROI | -0.351 | **0.036** | -0.626 | -0.012 |
|  | ERS Right ROI | -0.315 | 0.061 | -0.611 | 0.025 |
|  | ERS Frontal ROI | -0.429 | **0.009** | -0.636 | -0.168 |
| mFIS Psy | ERD Left ROI | 0.227 | 0.183 | -0.120 | 0.554 |
|  | ERD Right ROI | 0.215 | 0.207 | -0.130 | 0.544 |
|  | ERD Frontal ROI | 0.266 | 0.117 | -0.066 | 0.551 |
|  | ERS Left ROI | -0.196 | 0.252 | -0.504 | 0.112 |
|  | ERS Right ROI | -0.189 | 0.271 | -0.527 | 0.176 |
|  | ERS Frontal ROI | -0.306 | 0.069 | -0.617 | 0.028 |
| L 9-HPT | ERD Left ROI | 0.133 | 0.346 | -0.163 | 0.415 |
|  | ERD Right ROI | 0.069 | 0.628 | -0.221 | 0.335 |
|  | ERD Frontal ROI | 0.032 | 0.821 | -0.284 | 0.342 |
|  | ERS Left ROI | -0.278 | **0.046** | -0.527 | 0.010 |
|  | ERS Right ROI | -0.180 | 0.206 | -0.464 | 0.108 |
|  | ERS Frontal ROI | -0.179 | 0.205 | -0.461 | 0.134 |
| R 9-HPT | ERD Left ROI | 0.036 | 0.800 | -0.249 | 0.336 |
|  | ERD Right ROI | -0.012 | 0.934 | -0.268 | -0.272 |
|  | ERD Frontal ROI | 0.068 | 0.631 | -0.236 | 0.339 |
|  | ERS Left ROI | -0.250 | 0.074 | -0.511 | 0.023 |
|  | ERS Right ROI | -0.152 | 0.287 | -0.430 | 0.108 |
|  | ERS Frontal ROI | -0.186 | 0.186 | -0.464 | 0.093 |
| EDSS | ERD Left ROI | -0.092 | 0.593 | -0.388 | 0.211 |
|  | ERD Right ROI | -0.033 | 0.847 | -0.352 | 0.306 |
|  | ERD Frontal ROI | 0.125 | 0.466 | -0.205 | 0.441 |
|  | ERS Left ROI | -0.005 | 0.975 | -0.301 | 0.299 |
|  | ERS Right ROI | 0.039 | 0.820 | -0.287 | 0.368 |
|  | ERS Frontal ROI | -0.036 | 0.837 | -0.326 | 0.282 |
| MADRS | ERD Left ROI | 0.219 | 0.119 | -0.034 | 0.463 |
|  | ERD Right ROI | 0.140 | 0.326 | -0.157 | 0.427 |
|  | ERD Frontal ROI | 0.275 | 0.048 | -0.025 | 0.519 |
|  | ERS Left ROI | -0.167 | 0.236 | -0.461 | 0.155 |
|  | ERS Right ROI | -0.005 | 0.973 | -0.303 | 0.299 |
|  | ERS Frontal ROI | -0.249 | 0.075 | -0.505 | 0.075 |
| MSQOL Mh | ERD Left ROI | -0.254 | 0.148 | -0.580 | 0.097 |
|  | ERD Right ROI | -0.208 | 0.237 | -0.540 | 0.163 |
|  | ERD Frontal ROI | -0.444 | **0.009** | -0.713 | -0.135 |
|  | ERS Left ROI | 0.033 | 0.853 | –0.299 | 0.374 |
|  | ERS Right ROI | –0.113 | 0.523 | –0.460 | 0.221 |
|  | ERS Frontal ROI | 0.292 | 0.094 | –0.049 | 0.551 |
| MSQOL Ph | ERD Left ROI | –0.075 | 0.675 | –0.462 | 0.329 |
|  | ERD Right ROI | –0.078 | 0.661 | –0.420 | 0.334 |
|  | ERD Frontal ROI | –0.332 | 0.055 | –0.593 | 0.038 |
|  | ERS Left ROI | –0.147 | 0.408 | –0.517 | 0.216 |
|  | ERS Right ROI | –0.153 | 0.388 | –0.514 | 0.236 |
|  | ERS Frontal ROI | 0.141 | 0.426 | –0.262 | 0.499 |

**Supplementary Table 10.** Spearman correlation analyses between clinical measures and beta event-related desynchronization (ERD) and event-related synchronization (ERS) magnitude in the Left, Right and Frontal ROIs. Correlation coefficients (ρ) and uncorrected p-values are reported, with significant uncorrected p-values highlighted in bold. For reference, the family-wise Bonferroni-corrected significance threshold within each clinical scale or subscale is α = 0.0083.

**Abbreviations:** Fatigue Severity Scale (FSS); modified Fatigue Impact Scale, total score (mFIS Tot); modified Fatigue Impact Scale, cognitive subscale (mFIS Cog); modified Fatigue Impact Scale, physical subscale (mFIS Phy); modified Fatigue Impact Scale, psychosocial subscale (mFIS Psy); Nine-Hole Peg Test, left hand (L 9-HPT); Nine-Hole Peg Test, right hand (R 9-HPT); Expanded Disability Status Scale (EDSS); Montgomery-Åsberg Depression Rating Scale (MADRS); Multiple Sclerosis Quality of Life–54, mental health composite (MSQOL Mh); Multiple Sclerosis Quality of Life–54, physical health composite (MSQOL Ph); region of interest (ROI).

# **Supplementary Table 11 | Penalized logistic regression refit**

| **Predictor** | **β** | **OR** | **OR 95% CI (LCL–UCL)** |
| --- | --- | --- | --- |
| FA Left CST | -0.391 | 0.68 | 0.61 – 0.76 |
| ICF | -0.343 | 0.71 | 0.62 – 0.86 |
| FA-SLF-R | -0.341 | 0.71 | 0.62 – 0.84 |
| MSQOL-54-MH | -0.300 | 0.74 | 0.65 – 0.87 |
| Beta modulation Frontal ROI | -0.294 | 0.74 | 0.65 – 0.87 |
| CAU | -0.194 | 0.82 | 0.70 – 0.96 |
| Sex (Male vs Female) | -0.193 | 0.82 | 0.76 – 0.94 |
| FA Right CST | -0.239 | 0.79 | 0.69 – 0.91 |
| Disease Duration | -0.123 | 0.88 | 0.73 – 1.09 |
| MEP latency | -0.130 | 0.88 | 0.75 – 1.08 |
| FA Left SLF | -0.017 | 0.98 | 0.82 – 1.18 |

**Supplementary Table 11.** Penalized logistic regression refit reporting standardized regression coefficients (β) and odds ratios (ORs) for predictors of fatigue status. β denotes the standardized regression coefficient. Odds ratios are expressed per 1 standard deviation (SD) increase for continuous variables and as 1 vs. 0 for binary predictors. The outcome was coded as multiple sclerosis with fatigue (MS-F = 1) and multiple sclerosis without fatigue (MS-NF = 0). OR values < 1 indicate lower odds of fatigue. LCL, lower confidence limit; UCL, upper confidence limit.
Abbreviations: Fractional anisotropy (FA); left corticospinal tract (Left CST); right corticospinal tract (Right CST); superior longitudinal fasciculus (SLF); right superior longitudinal fasciculus (SLF-R); intracortical facilitation (ICF); Multiple Sclerosis Quality of Life–54, mental health composite (MSQOL-54 MH); beta modulation depth (beta modulation); frontal region of interest (Frontal ROI); caudate nucleus volume (CAU); motor evoked potential latency (MEP latency); odds ratio (OR); lower confidence limit (LCL); upper confidence limit (UCL).
